# Merkel Cell Polyomavirus Encodes Circular RNAs (circRNAs) Enabling a Dynamic circRNA/microRNA/mRNA Regulatory Network

**DOI:** 10.1128/mBio.03059-20

**Published:** 2020-12-15

**Authors:** Bizunesh Abere, Hongzhao Zhou, Jinghui Li, Simon Cao, Tuna Toptan, Adam Grundhoff, Nicole Fischer, Patrick S. Moore, Yuan Chang

**Affiliations:** a Hillman Cancer Center, Cancer Virology Program, University of Pittsburgh, Pittsburgh, Pennsylvania, USA; b Department of Microbiology and Molecular Genetics, University of Pittsburgh, Pittsburgh, Pennsylvania, USA; c School of Medicine, Tsinghua University, Haidian District, Beijing, People’s Republic of China; d Department of Biological Sciences, University of Pittsburgh, Pittsburgh, Pennsylvania, USA; e Department of Pathology, University of Pittsburgh, Pittsburgh, Pennsylvania, USA; f Institute of Medical Virology, University Hospital Frankfurt am Main, Goethe University, Frankfurt am Main, Germany; g Research Group Virus Genomics, Heinrich Pette Institute, Leibniz Institute for Experimental Virology, Hamburg, Germany; h Institute for Medical Microbiology, Virology and Hygiene, University Medical Center Hamburg-Eppendorf, Hamburg, Germany; Columbia University College of Physicians & Surgeons

**Keywords:** Merkel cell carcinoma, Merkel cell polyomavirus, T antigen, circular RNA, micro RNA, noncoding RNA, polyomaviruses

## Abstract

Viral noncoding RNAs have acquired increasing prominence as important regulators of infection and mediators of pathogenesis. Circular RNAs (circRNAs) generated by backsplicing events have been identified in several oncogenic human DNA viruses. Here, we show that Merkel cell polyomavirus (MCV), the etiologic cause of ∼80% of Merkel cell carcinomas (MCCs), also expresses circular RNAs. By RNase R-resistant RNA sequencing, four putative circRNA backsplice junctions (BSJs) were identified from the MCV early region (ER). The most abundantly expressed MCV circRNA, designated circMCV-T, is generated through backsplicing of all of ER exon II to form a 762-nucleotide (nt) circular RNA molecule. Curiously, circMCV-T, as well as two other less abundantly expressed putative MCV circRNAs, overlaps in a complementary fashion with the MCV microRNA (miRNA) locus that encodes MCV-miR-M1. circMCV-T is consistently detected in concert with linear T antigen transcripts throughout infection, suggesting a crucial role for this RNA molecule in the regulatory functions of the early region, known to be vital for viral replication. Knocking out the hairpin structure of MCV-miR-M1 in genomic early region expression constructs and using a new high-efficiency, recombinase-mediated, recircularized MCV molecular clone demonstrates that circMCV-T levels decrease in the presence of MCV-miR-M1, underscoring the interplay between MCV circRNA and miRNA. Furthermore, circMCV-T partially reverses the known inhibitory effect of MCV-miR-M1 on early gene expression. RNase R-resistant RNA sequencing of lytic rat polyomavirus 2 (RatPyV2) identified an analogously located circRNA, stipulating a crucial, conserved regulatory function of this class of RNA molecules in the family of polyomaviruses.

## Abstract (precis)

Covalently closed circular RNAs were recently described in the human DNA tumor viruses Epstein-Barr virus (EBV), Kaposi’s sarcoma-associated herpesvirus (KSHV), and human papillomavirus (HPV). Here, we show that MCV, another DNA tumor virus, generates circRNAs from its early regulatory region in concert with T antigen linear transcripts.

## INTRODUCTION

Merkel cell polyomavirus (MCV) causes ∼80% of Merkel cell carcinoma (MCC) tumors (1). MCV is a nonenveloped, double-stranded DNA virus whose genome is divided into two bidirectionally oriented and sequentially expressed transcriptional units: the early region (ER) and the late region (LR). Generated through alternative splicing, MCV ER transcripts express tumor antigen (T-Ag) proteins, including large T antigen (LT), small T antigen (sT), 57,000-molecular-weight T antigen (57kT), and the alternative large T antigen open reading frame (ALTO) (2–4). Some of these ER-encoded proteins have characterized functions essential for MCV replication and MCC oncogenesis (4–17). The MCV LR produces transcripts that define structural proteins VP1 and VP2, necessary for MCV virion production (18).

MCV also generates noncoding RNAs (ncRNAs). A microRNA (miRNA) precursor is transcribed from the negative strand of the ER (miR-M1) and gives rise to two mature miRNAs, MCV-miR-M1-5P and MCV-miR-M1-3P (2, 19). miRNAs are ∼22-nucleotide (nt) molecules derived from precursor RNA, processed by Drosha and Dicer. Incorporated into the RNA-induced silencing complex (RISC), miRNA results in mRNA degradation or translation inhibition of its target transcript (20, 21). MCV-miR-M1 shows perfect complementary sequence identity to a region in the early gene transcripts, resulting in the degradation of these transcripts (19). By regulating LT transcripts, MCV-miR-M1 inhibits MCV replication and thus contributes to the maintenance of the MCV genome as episomes in the infected host cell (2, 19).

Circular RNAs (circRNAs) are 3′-to-5′ covalently cyclized RNA molecules that are produced through a backsplicing mechanism (22–25). Because of their circular conformation, circRNAs are less susceptible to exonuclease activity, resulting in their increased stability compared to that of their linear counterparts (22, 23, 26, 27). When circRNAs were first identified in the early 1990s, they were considered a “mis-splicing” product (28). However, in the past decade, circRNAs have been found to be critical cellular regulators during development and disease, with the potential to be used as biomarkers as a result of their differential tissue- and disease-specific expression profiles (29–34). Several broad functions have been ascribed to circRNAs. They have been shown to act as miRNA sponges (29, 33, 35). They can also regulate RNA binding protein (RBP)/RNA interactions (36–38) or code for proteins through cap-independent translation (39, 40). circRNAs are not limited to eukaryotic cells. Virus-encoded circRNAs were described in 2018 in the human herpesviruses Epstein-Barr virus (EBV) and Kaposi’s sarcoma-associated herpesvirus (KSHV) (41–44) and then subsequently in 2019 in human papillomavirus (HPV) (45). Attributable functions of most of these virus-encoded circRNAs are still undefined. However, HPV-encoded circE7 has been reported to be translated into an E7 oncoprotein (45), and the EBV-encoded circLMP2A may induce stemness in EBV-associated gastric cancer (46). It has not previously been determined whether the human tumor virus MCV, in like fashion to these oncogenic DNA viruses, can encode circRNAs.

In this study, we identify circRNA backsplice junctions (BSJs) in polyomaviruses by RNase R+ sequencing. For MCV, we characterized the expression of the most abundant MCV circRNA (designated circMCV-T) in MCV genome-transfected 293 cells as well as in MCC tumor-derived cell lines that have MCV monoclonally integrated into the host genome. These two conditions represent two different outcomes of MCV infection in human cells: that of active viral DNA replication/infection and that of replication-deficient, virus-induced transformation. circMCV-T is consistently detectable in concert with ER linear mRNA transcripts as well as with MCV miRNA when expressed from a fully intact, nonintegrated viral genome. However, with replication-deficient integrated genomes, which occur in MCV-positive MCC cell lines, circMCV-T expression is inconsistently detectable; it is present in WaGa and CVG-1 but not in MKL-1, MKL-2, or MS-1 cells. Using MCV ER expression vectors as well as an efficient recombinase-induced DNA circularization minicircle (mc) technology to produce an MCV molecular clone, we find that the expression pattern of circMCV-T is inversely related to MCV-miR-M1 levels, and its exogenous expression stabilizes the mRNA of MCV early transcripts.

## RESULTS

### Identification of Merkel cell polyomavirus-encoded circRNAs by RNase R+ RNA sequencing.

**FIG 1:**
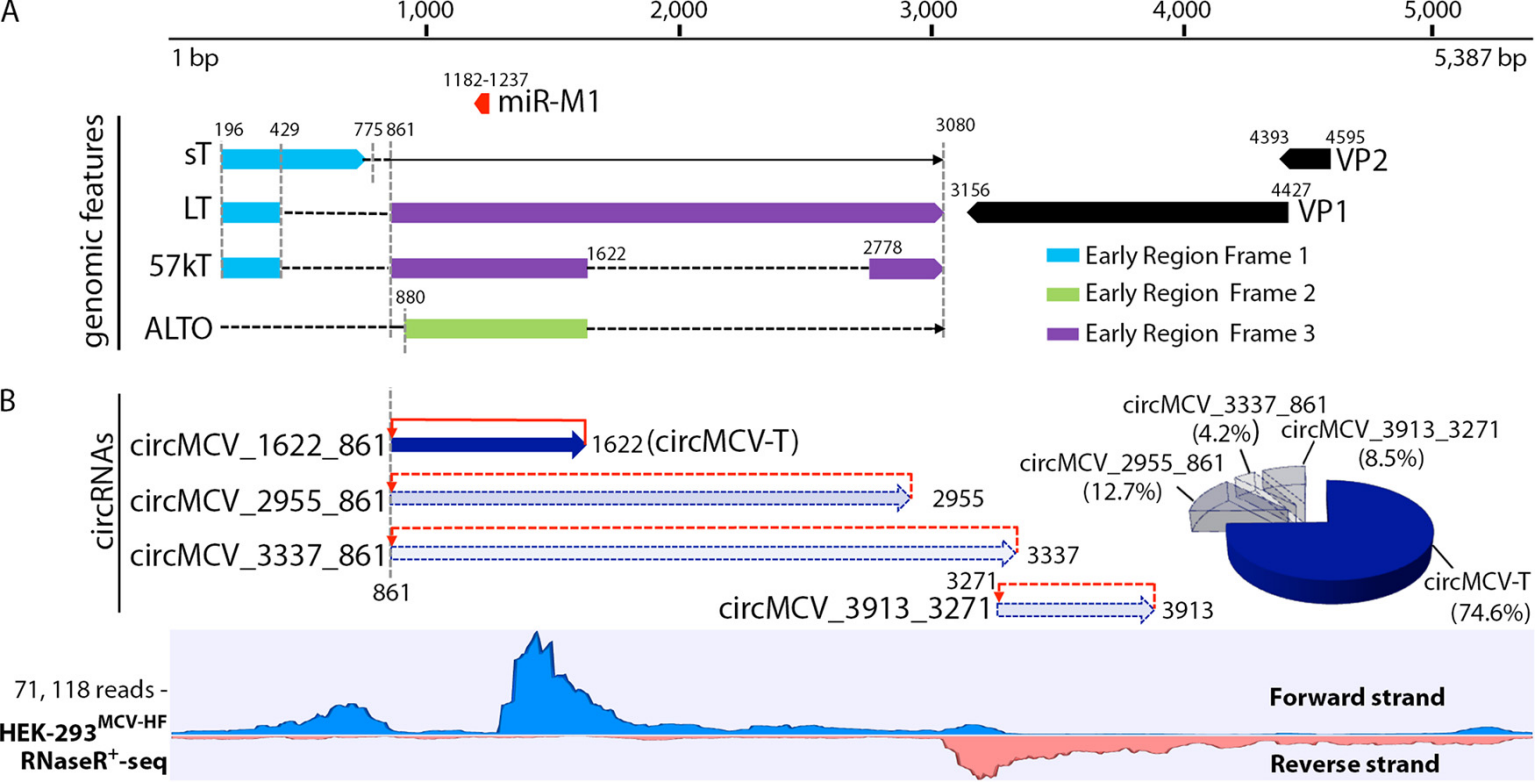
Identification of MCV-encoded circRNAs by RNase R+ RNA sequencing. (A) Schematic representation of the MCV genome organization. Structures of known MCV early transcripts are shown. Positions of splice donor or acceptor sites are shown by vertical dashed lines and/or numbers. Known mRNAs are represented with solid arrows, while protein products are shown as boxes. Dashed lines show sequences that are spliced out of the indicated RNA transcript. (B, bottom) RNase R+ RNA sequence coverage of MCV-HF in transfected 293 cells after 5 days of transfection. Sequence reads from the forward strand (blue) and reverse strand (red) are shown. (Top) Schematic representation of the four putative MCV-encoded circRNA backsplice junctions (BSJs) mapped to the MCV-HF genome. Red arrows show backsplicing directions. The percentage of each putative circRNA BSJ count from the ER is shown in a pie chart (right). The sequencing result is from one experiment.

To investigate whether polyomaviruses, specifically MCV, can encode circRNAs, RNA sequencing was performed on RNA isolated from two models of MCV infection: 293 cells transfected with an *in vitro*-recircularized wild-type (wt) MCV-HF genome (GenBank accession no. JF813003) (7) and CVG-1 or MS-1 MCC-derived cell lines with an integrated viral genome and ER polymorphisms that abolish genome replication. We treated extracted RNA with RNase R to deplete linear transcripts and to enrich for circular forms of RNA. Since our goal for sequencing was to identify circular RNA molecules, we did not process samples not treated with RNase R, nor did we perform sequencing repeats. MCV circRNA backsplice junction (BSJ) candidates were identified by the CIRI2 circRNA prediction algorithm (47) using the consensus genome MCV-HF (accession no. JF813003) as a reference. The structure and arrangement of MCV early T antigen and late transcripts as well as miR-M1 are shown in Fig. 1A. Total RNase R-resistant reads and reads mapping to the MCV genome for each cellular model of MCV infection are shown in Table S1 in the supplemental material. In comparison to the replicating model of MCV-HF-transfected 293 cells, those of the MCC cell lines MS-1 and CVG-1 displayed at least 2 orders of magnitude-lower numbers of RNase R-resistant viral reads despite the total numbers of reads being comparable between the two models. This is in agreement with a previous report where the numbers of viral sequence reads from poly(A) sequencing of MCC-derived cell lines MKL-1 and WaGa were significantly lower than for PFSK-1 cells transfected with MCVsyn, a circularized, wild-type genome identical to MCV-HF (2). A total of seven distinct MCV BSJs were detected from MCV-HF-transfected 293 cells by RNase R+ sequencing (Table S2), while there were no BSJ reads detected from the MCC-derived cell lines CVG-1 and MS-1. This is consistent with the low number of viral transcripts produced in these cell lines, which have contracted viral transcriptional programs compared to those of replication-competent viral genomes in the 293 transfection model. The contribution of total combined positive MCV circRNA BSJ reads to the total RNase R+ sequenced reads from the MCV early region (ER) is very low (<0.019 reads per million [RPM]) (Table S2) compared to the circRNA reads from the EBV (∼37 to 440 RPM for circBARTs) and KSHV (∼200 RPM for circv-IRF4) genomes (41–43). We observed depletion of sequence coverage corresponding to the 5′-most part of the putative circMCV-T coding region. The sequencing coverage plot also displayed RNase R-resistant peaks corresponding to regions in the first exon of LT and sT, as well as parts of the late genes (Fig. 1B, bottom).

10.1128/mBio.03059-20.4 TABLE S1 Summary of RNase R+ RNA-seq reads obtained from MCC cell lines, MCV-HF-transfected 293 cells, and RatPyV2-infected rat parotid gland. Download Table S1, PDF file, 0.04 MB. Copyright © 2020 Abere et al. 2020 Abere et al. This content is distributed under the terms of the Creative Commons Attribution 4.0 International license.

10.1128/mBio.03059-20.5 TABLE S2 Sequenced BSJs from MCV-HF. Download Table S2, PDF file, 0.04 MB. Copyright © 2020 Abere et al. 2020 Abere et al. This content is distributed under the terms of the Creative Commons Attribution 4.0 International license.

Out of the seven different BSJs found in the MCV-HF-transfected 293 cells, four were detected from the viral ER (Fig. 1B and Table S2). Among these four putative circRNA BSJs, circMCV_1622-861 or circular MCV-T (named circMCV-T) was the most abundant, comprising 74.6% of the total 71 BSJs detected from the forward strand. The circMCV-T BSJ is generated through backsplicing of the 3′ donor at nt 1622 (2–4) to an upstream 5′ acceptor at nt 861, circularizing the entire exon II of T-Ag transcripts to produce a 762-nt circRNA (Fig. 1A and B). Although the splicing coordinates for circMCV-T correspond to those of exon II generated by canonical forward splicing to produce the 57kT transcript, in fact, all known T-Ag transcripts (LT, sT, 57kT, and ALTO) can be affected by the usage of these splice sites because the annotated splice acceptor and donor sites are required for the splicing of all naturally occurring alternative transcripts (Fig. 1 A). Two other low-abundance putative circRNA BSJs from the ER, circMCV_2955-861 (12.7% of 71 BSJs) and circMCV_3337-861 (4.2% of 71 BSJs), similarly use the same canonical splice acceptor at nt 861 but differ in their splice donor sites located at nt 2955 and 3337, respectively. These splice donor sites have not been previously annotated to be recruited in forward splicing events (Fig. 1B and Table S1); however, these sites contain a splicing donor sequence (/GTAA) similar to that of circMCV-T. A fourth low-abundance BSJ (circMCV_3913-3271 [8.5%]) is also detected from the forward strand. Unlike the three circRNAs that are same-stranded in relation to T-Ag coding transcripts and share the same backsplice acceptor coordinate, this fourth BSJ has unique splicing coordinates and is complementary to the VP1 coding transcripts (Fig. 1B and Table S1).

Three of the seven MCV BSJ reads were detected from the late region (LR) (Table S1). These reads all contain a putative common splice donor at nt 1142, which has previously been identified as a low-frequency splice site (2), coupled with an acceptor site at nt 4642, used by the VP1 transcript, nt 5119, used by the VP2 transcript, or nt 5308, another low-frequency acceptor site (2). However, these BSJ-simulating reads may not actually represent authentic circRNA-forming backsplicing events; instead, these reads are likely to arise during leader-to-leader forward splicing of multigenomic LR RNA precursors formed by the action of RNA polymerase II (Pol II) multiply circuiting the viral genome. This process is believed to account for the accumulation of late viral mRNA transcripts during late stages of viral replication (48–50).

### MCV circRNA validation and characterization.

**FIG 2:**
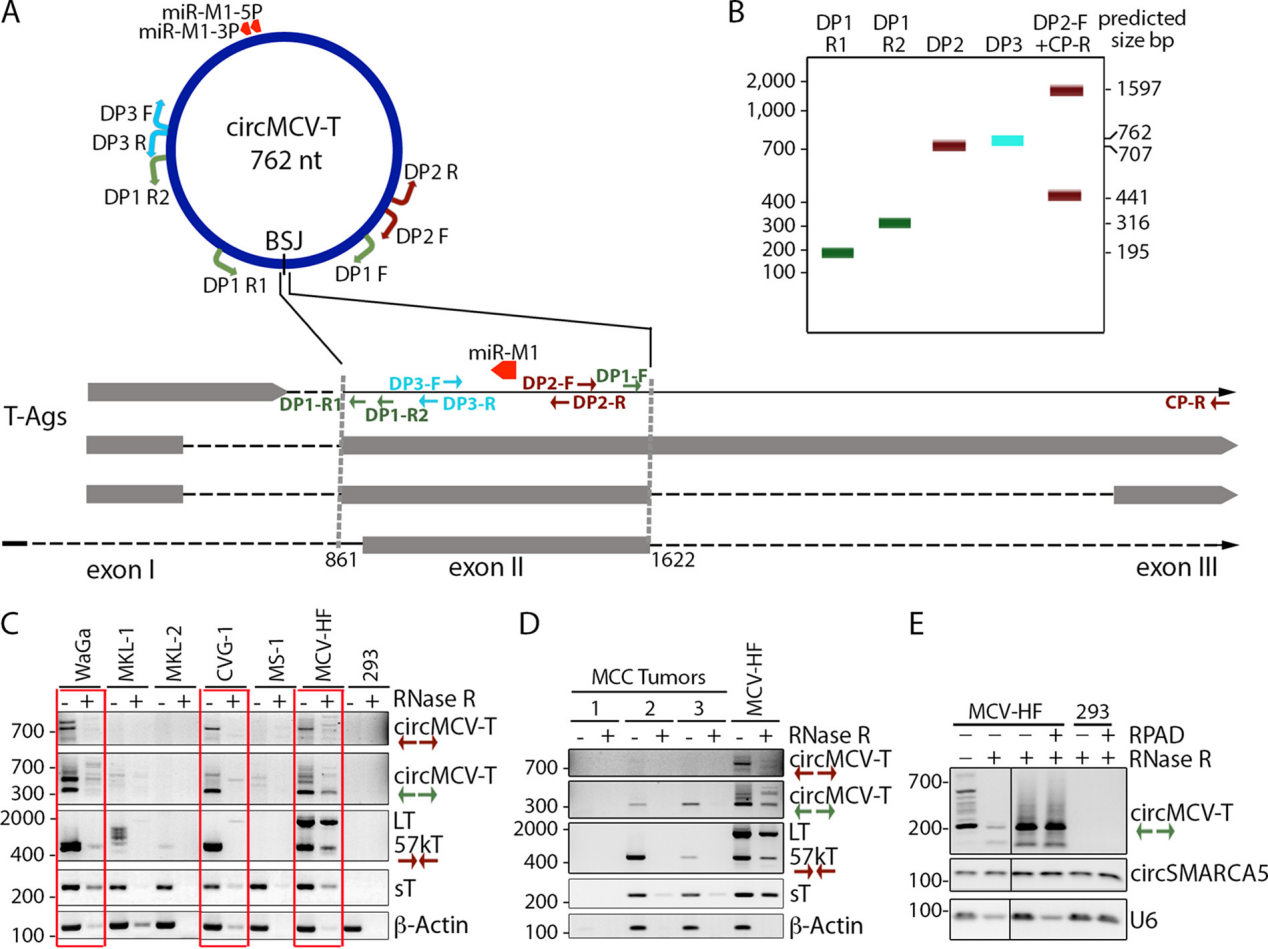
Validation and characterization of circMCV-T in MCC-derived cell lines and primary tumor tissues. (A) Schematic representation of MCV T-Ag transcripts and circMCV-T. Divergent primer pairs used for circMCV-T RT-PCR are shown with color codes. (B) Schematic representation of expected PCR products using each primer pair in panel A. (C and D) Expression profiles of circMCV-T in MCC cell lines (Waga, MKL-1, MKL-2, CVG-1, MS-1) and MCV genome (MCV-HF)-transfected 293 cells (C) and in patient-derived tumor samples (MCC 1 to 3) (D) in the presence and absence of RNase R treatment are detected by RT-PCR using the indicated divergent primer pairs (DP) for circMCV-T and conventional convergent primer pairs for linear transcripts (sT, 57kT, LT, and β-actin). Untransfected 293 cells were used as a negative control, and linear RNAs were used as an RNase R digestion control. See Table 2 for the location of each primer on the MCV genome. (E) RPAD was performed on RNase R-treated RNA from 293 cells transfected with the MCV-HF genome for 5 days. The cellular circRNA SMARCA5 is used as a control that should not be depleted after RPAD treatment, while U6 was used as linear RNA control that should be depleted by RPAD. Each result represents a single experiment. Numbers at the left of the blots are molecular sizes in base pairs.

The putative circMCV-T is the most abundant BSJ, comprising 74.6% of the total 71 BSJs reads from the early region of the MCV genome (Fig. 1B and Table S2). Therefore, further characterization and analysis were directed at circMCV-T. Divergent primer (DP) pairs, amplifying either fragments or the full circle spanning the BSJ, were designed for reverse transcription (RT)-PCR analysis (Fig. 2A). Exact nucleotide positions of primers are provided (see Table 2). Because of the overlapping nature of the three putative circRNAs from the MCV ER, all DP pairs have the potential to amplify each of these circRNAs, although differing in amplification product sizes. Primer pairs DP1 (DP1.F coupled either with reverse primer DP1.R1 or with DP1.R2) are calculated to produce bands of 195 or 316 bp, respectively, from circMCV-T. The DP2 primer pair will produce a 707-bp product, while the back-to-back divergent primer pair DP3 will produce a 762-bp product, representing the entire circMCV-T (Fig. 2B). RT-PCR using primer pairs DP2 and DP1.R2 detected both the large and small fragments (at the predicted sizes) of circMCV-T in WaGa (51), CVG-1 (52), and 293 cells transfected with MCV-HF in non-RNase R-treated samples (Fig. 2C, red outline), while no products of predicted sizes were detected from MKL-1, MKL-2, and MS-1 cells (Fig. 2C). An additional PCR product with a larger size was also detected in WaGa cells for both the DP2 and DP1.R2 divergent primer pairs. Sequencing identified them to represent circMCV_2955_861 with the 57kT intron spliced out. As seen previously, linear LT and 57kT transcript levels vary widely between cell lines infected with different strains of MCV; however, linear viral sT transcript levels are relatively constant. Therefore, to confirm RNA quality and RNase R treatment efficiency, sT transcript levels along with cellular β-actin transcript levels were used as controls. The absence of detectable circMCV-T products from MKL-1 cells is consistent with the absence of the splice donor at nt 1622 due to a naturally occurring 46-nucleotide deletion (nt 1612 to 1657) in this particular integrated viral genome strain (1, 4). The MKL-2 viral strain contains a 2-bp deletion at nt 3082 to 3083, while the MS-1 viral strain has a 40-bp deletion at nt 1912 to 1951 (1, 4). Genomic polymorphisms in these two virus strains leave the circMCV-T splice donor (nt 1622) and the splice acceptor (nt 861) intact and, therefore, should not preclude the formation of circMCV-T. However, other as-yet-undefined sequence-related features might affect splicing in these genomes. Remarkably, the presence of circMCV-T expression in these MCC-derived cell lines mirrors that of 57kT transcript expression (Fig. 2C). PCR using an intron-spanning convergent primer pair (DP2-F and CP-R) results in two PCR products corresponding to LT (1597 bp) and 57kT (441 bp). The larger fragment representing the LT transcript is observed only in the positive-control MCV-HF-transfected 293 cells, while the smaller fragment corresponding to the 57kT transcript can be detected only in WaGa and CVG-1 cells, not in MKL-1, MKL-2, or MS-1 cells. As with circMCV-T, the deletions in MKL-2 and MS-1 strains also should not affect 57kT splicing.

BSJ-containing fragments of circMCV-T were also detected from two out of three MCC patient-derived tumor samples tested (MCC-2 and MCC-3). In the MCC-1 sample, from which no circMCV-T was found, linear virus (LT, 57kT, sT) and a cellular control (β-actin) were also not detectable, suggesting that the quality of the extracted RNA was inadequate (Fig. 2D). The DP1.R2 primer pair detected the predicted 316-bp product in tumor samples with intact RNA as well as MCV-HF-infected 293 cells. The DP2 primer pair, which can amplify almost the entire circMCV-T, detected the predicted 707-bp band only from the MCV-HF-infected 293 cells and not from MCC tumor samples. The PCR products from Fig. 2C and D were confirmed to contain circMCV-T BSJs by sequencing. The three low-abundance BSJs from the RNase R+ sequencing are not reliably detectable in naturally infected tissues or in cell lines by RT-PCR. Their functional significance is difficult to assess.

Unlike with circRNAs, whose nature is reportedly exonuclease resistant (22, 27), the majority of circMCV-T is RNase R sensitive in a fashion similar to that of viral and cellular linear transcripts (Fig. 2C and D). Therefore, to further confirm that completely cyclized circMCV-T forms occur, we applied RNase R treatment, polyadenylation, and poly(A)^+^ RNA depletion (RPAD) (53) on RNA from MCV-HF-transfected cells. The RT-PCR results from RPAD-processed and -unprocessed samples (Fig. 2E) show no further depletion of circMCV-T from RNase R-treated samples after RPAD (Fig. 2E, top, 3rd and 4th lanes), indicating the presence of true circular forms of circMCV-T. Cellular circSMARCA5 was assayed as a control that is not depleted by RNase R and RPAD, while the linear RNA U6 was used as a control that can be depleted by RPAD.

### circMCV-T detection *in situ*.

**FIG 3:**
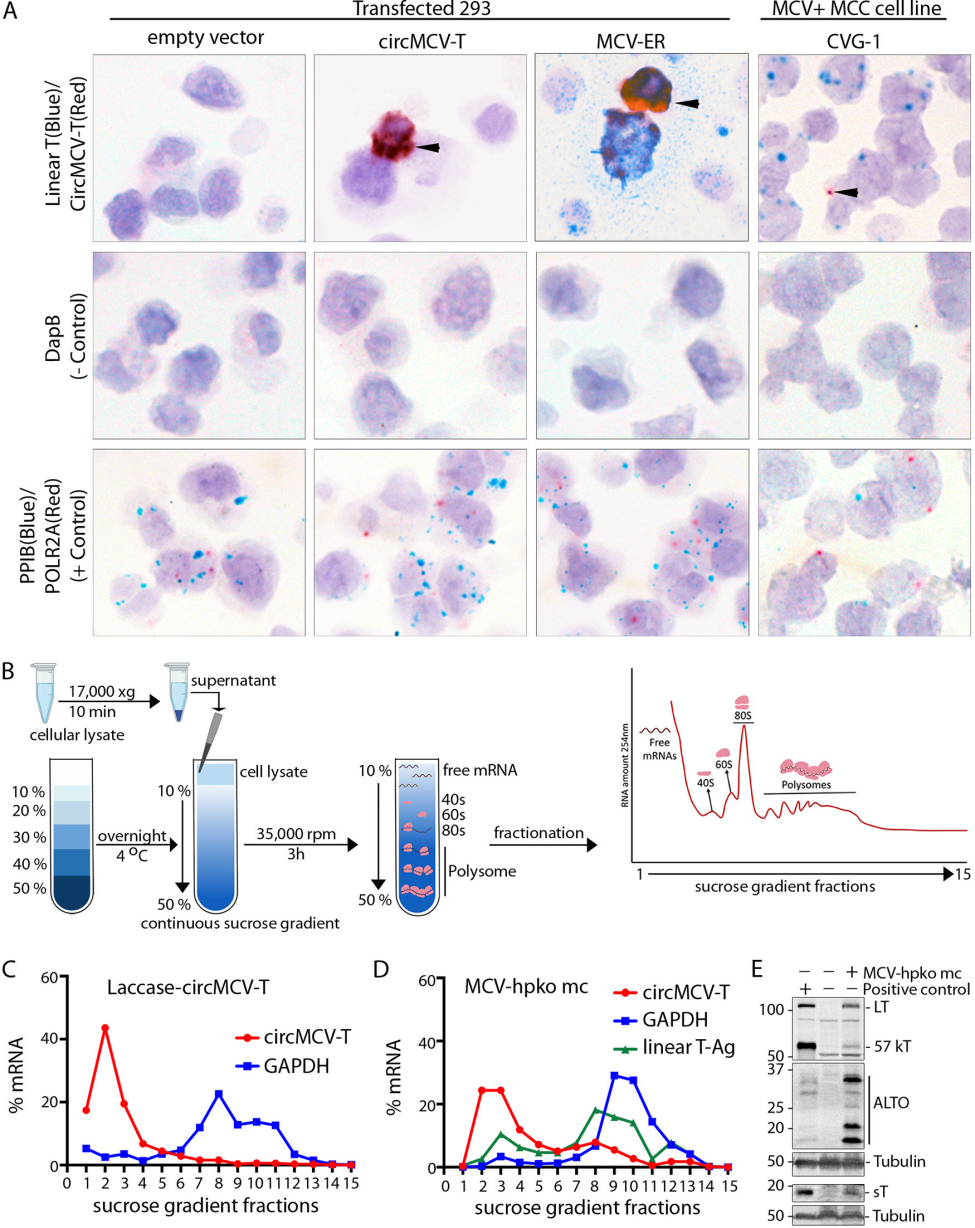
*In situ* detection and polysome fractionation of circMCV-T. (A) Detection of MCV circMCV-T BSJ by BaseScope *in situ* hybridization. Representative images from 293 cells transfected with an expression vector for circMCV-T or MCV ER (early region) are shown. CVG-1, an MCV^+^ MCC cell line with circMCV-T detectable by RT-PCR, shows an abundance of linear T-Ag transcripts (blue) compared with circMCV-T transcripts (red). 293 cells transfected with an empty vector were used as a negative control. The red signal represents the detection of circMCV-T, the cellular control POLR2A, or the bacterial negative-control gene DapB, while the blue signal represents staining for linear T-Ag RNA or the cellular control gene *PPIB*. Images were originally acquired at a ×40 magnification. (B) Polysome fractionation assay workflow created using BioRender.com. (C and D) RT-qPCR of circMCV-T (red), linear T-Ag (green), and GAPDH (blue) transcripts performed on polysome fractions from (C) pLaccase-circMCV-T-transfected (no linear T-Ag is produced from this expression construct and was therefore not assessed) and (D) MCV-HF-hpko mc-transfected 293 cells. (E) Western blot of MCV-encoded proteins from the MCV early (LT, 57KT, ALTO, and sT) regions from MCV-HF-mc-transfected 293 cells on which polysome fractionation is depicted in panel C with α-tubulin used as a protein internal control. Experiments whose results are represented in panels C, D, and E were each performed at least two times.

To investigate the presence of circMCV-T *in situ*, BaseScope RNA hybridization was applied to cell pellet arrays comprised of CVG-1 and 293 cells transfected with either the MCV ER expression construct, a pLaccase-circMCV-T construct, or an empty pLaccase vector control. A BSJ-spanning 1z probe (red) to circMCV-T was detected in all samples except empty-vector-transfected negative-control cells (Fig. 3A). The 2zz probe (blue) to linear T-Ag at a position outside the circRNA-coding region was detected in MCV ER-transfected 293 cells and in the MCC-derived CVG-1 cell line but not in pLaccase-circMCV-T- or pLaccase empty-control-transfected cells. As expected, the highest level of circMCV-T expression was found in pLaccase-circMCV-T and MCV ER control-transfected cells, where the red signal manifested as extensive clumps obliterating cytologic details rather than as individual dots. Signals detected as clumps occur under conditions of high levels of ectopic RNA expression and cannot be quantified. Furthermore, the BaseScope *in situ* hybridization protocol is much longer and more harsh on tissue section morphology; therefore, loss of crispness (e.g., nuclear details and hematoxylin counterstaining, for example) is not unexpected and cannot be construed to be an effect of circMCV-T expression. The signal and number of cells positive are almost entirely dependent on transfection efficiency in the controls. However, in CVG-1 cells, circMCV-T expression is observed as red dots of low occurrence in 4.7% of 402 cells counted versus 85.3% for linear T-Ag (Table S3). Cellular linear transcripts PPIB (blue) and POLR2A (red) were used as controls for RNA quality. A probe against the bacterial DapB gene is used as a negative control.

10.1128/mBio.03059-20.6 TABLE S3 circMCV-T BaseScope *in situ* hybridization detection in CVG-1 cells. Download Table S3, PDF file, 0.04 MB. Copyright © 2020 Abere et al. 2020 Abere et al. This content is distributed under the terms of the Creative Commons Attribution 4.0 International license.

### circMCV-T is unlikely to be translated into a protein product.

Some circRNAs have been shown to code for proteins through internal ribosome entry site (IRES)- or *N*^6^-methyladenosine (m^6^A)-mediated, 5′-cap-independent translation initiation (39, 40, 45). To test whether circMCV-T has the potential to code for a protein product, we performed polysome fractionation (Fig. 3B) on 293 cells transfected with the pLaccase-circMCV-T construct or MCV-HF mc molecular clone with a mutation to knock out miR-M1 expression (MCV-hpko mc). The miR-M1 hpko mutation has previously been shown to increase viral replication (2). Polysome fractionation showed that while the positive-control linear RNA GAPDH (glyceraldehyde-3-phosphate dehydrogenase) is found associated with polysomes in fractions 7 through 12, circMCV-T is detected only in the earlier fractions that do not contain polysomes in cells transfected with pLaccase-circMCV-T (Fig. 3C). In cells transfected with MCV-hpko mc, the slight increase in detection around fraction 8 is of undetermined significance (Fig. 3D). A Western blot control for viral protein expression from MCV-hpko mc is shown in Fig. 3E

We also assayed for the protein-coding capacity of circMCV-T by Western blot analysis using two antibodies with epitope targets covering two frames of possible protein translation in exon II: the CM2B4 antibody, traditionally used to detect LT and 57kT (ER frame 3), and the CM7B1 antibody, which detects ALTO (ER frame 2) (Fig. S1.A). The third frame (ER frame 1) contains a single start codon and multiple stop codons that can theoretically code for a very small protein product of ∼9 kDa. With this caveat and within the limitations of immunoblotting sensitivity, and as well as with the specific binding efficacies of these antibodies, we were unable to detect a protein product from 293 cells transfected with pLaccase-circMCV-T (Fig. S1.B).

10.1128/mBio.03059-20.1 FIG S1 Assay for the circMCV-T-encoded protein product. (A) Schematic showing all three potential open reading frames present within circMCV-T aligned to the consensus MCV-HF genome (GenBank accession no. JF813003). Only the +1 and +2 reading frames encode methionine amino acids, and only the +1 reading frame encodes stop codons. The only peptide potentially encoded by the +1 frame has a size of 9.1 kDa. (B) 293 cells transfected with plasmids expressing circ-T-Ag and blotted with antibodies to epitopes within the +2 and +3 reading frames show no protein translation to the limit of detection. The same membrane was incubated with both antibodies, with CM7B1 blotting preceding CM2B4 blotting. Download FIG S1, TIF file, 1.5 MB. Copyright © 2020 Abere et al. 2020 Abere et al. This content is distributed under the terms of the Creative Commons Attribution 4.0 International license.

### The absence of miR-M1 expression increases the MCV circMCV-T level.

**FIG 4:**
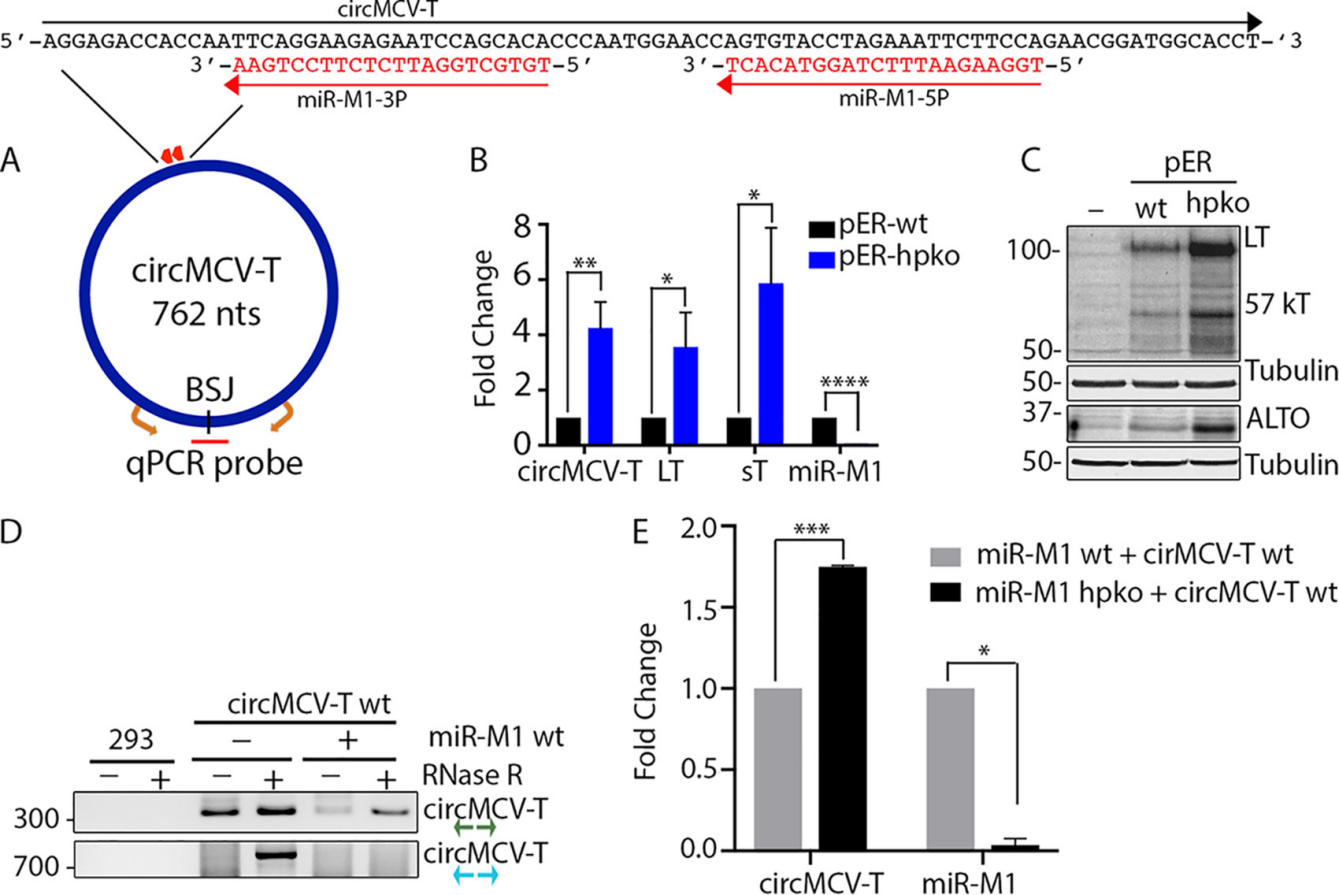
The presence of MCV miR-M1 decreases circMCV-T levels. (A) Schematic representation of MCV circMCV-T and miR-M1 features. Mature MCV-miR-M1-5P and -3P binding sites are shown as red arrows, and sequence features at the miRNA binding site are represented at the top. Divergent primers (orange) and a BSJ spanning TaqMan probe (red) are used for circMCV-T qPCR amplification. (B) Expression of circMCV-T and T-Ag RNA from MCV early region expression constructs containing either a wt or hpko mutation for MCV miR-M1. (C) Expression of T antigen proteins (LT, 57KT, and ALTO) from panel B. (D) RT-PCR detection of circMCV-T expressed from a pLaccase-circMCV-T construct in the presence and absence of MCV-miR-M1 using the indicated divergent primer pairs 48 h after transfection of 293 cells. (E) RT-qPCR quantification of circMCV-T levels from a pLaccase-circMCV-T construct in the presence of either wt or hpko mutant MCV miR-M1 48 h posttransfection of 293 cells. Results in panels C and D are representative of three independent experiments. *C_T_* values were normalized to RNase P (B) or GAPDH (E) *C_T_* values, and samples were normalized to pER-wt (B)- or wt miR-M1 (E)-transfected cells to calculate fold change. Error bars in panels B and E represent means ± standard deviations (SD) from three independent experiments. Statistical analysis was performed on ΔΔ*C_T_* values using an unpaired *t* test. *, *P* < 0.05; **, *P* < 0.01; ***, *P* < 0.001; ****, *P* < 0.0001.

The MCV ER contains the coding sequence for MCV miR-M1, complementary to the T-Ag coding region of the viral genome. The resulting mature miRNA seed sequences miR-M1-5P and -3P can bind the reverse strand with 100% complementarity (Fig. 1A and 4A). This complete complementarity results in the degradation of linear T antigen transcripts, leading to suppression of viral replication (2). Because circMCV-T also has the MCV miRNA binding sites, we hypothesize that the RNase R sensitivity of circMCV-T is likely to be due to its linearization by miR-M1, leading to the apparent low number of BSJ reads detected from the MCV genome (Table S2). To test this, we compared the levels of circMCV-T as well as of linear LT and sT transcripts from the MCV ER construct containing the wild-type (wt) sequence for miR-M1 or containing mutations in the pre-miR-M1 seed sequence resulting in a hairpin knockout (hpko) that abolishes mature MCV miR-M1 expression (2). As previously described (2), we saw an increase in linear sT and LT transcripts in the hpko mutant MCV ER compared to levels in the wt construct; circMCV-T levels were also detected at a higher level in the hpko mutant MCV ER, as detected by quantitative RT-PCR (RT-qPCR) using a BSJ-spanning TaqMan probe and a pair of divergent primers on either side of the BSJ (Fig. 4A and B). In contrast, miR-M1 is undetectable in the hpko mutant, unlike in the wt construct, as detected by stem-loop RT-PCR analysis. As with previous reports (2), protein expression levels of LT, 57 KT, and ALTO are also increased as a result of the miR-M1 hpko mutation (Fig. 4C). To better understand the functional interaction of circMCV-T with miR-M1, we cotransfected pLaccase-circMCV-T with a wild-type or hpko mutant miR-M1 expression vector and analyzed circMCV-T levels by RT-PCR and qPCR. Note that the wt pLaccase-circMCV-T expression construct can potentially express miR-M1 from the opposite-strand DNA sequence due to the presence of an miR-M1 promoter described previously (2). Unlike with cells not transfected with miR-M1, cells cotransfected with the wt miR-M1 expression vector exhibited decreased circMCV-T levels after RNase R treatment, as detected by the DP1 primer pair (Fig. 4D, top). The presence of miR-M1 completely abolished the detection of the full-length circMCV-T using a back-to-back primer pair (DP3) that can amplify the entire 762-bp circle. In the absence of miR-M1 expression, circMCV-T is enriched after RNase R treatment (Fig. 4D, bottom). Additionally, expression of miR-M1 from a wt expression vector depleted circMCV-T levels compared to those after cotransfection of an hpko mutant miR-M1 construct (Fig. 4E). Together, these results support the notion that miR-M1 mediates linearization of circMCV-T, resulting in its RNase R sensitivity.

### Role of circMCV-T in early gene expression and viral replication.

**FIG 5:**
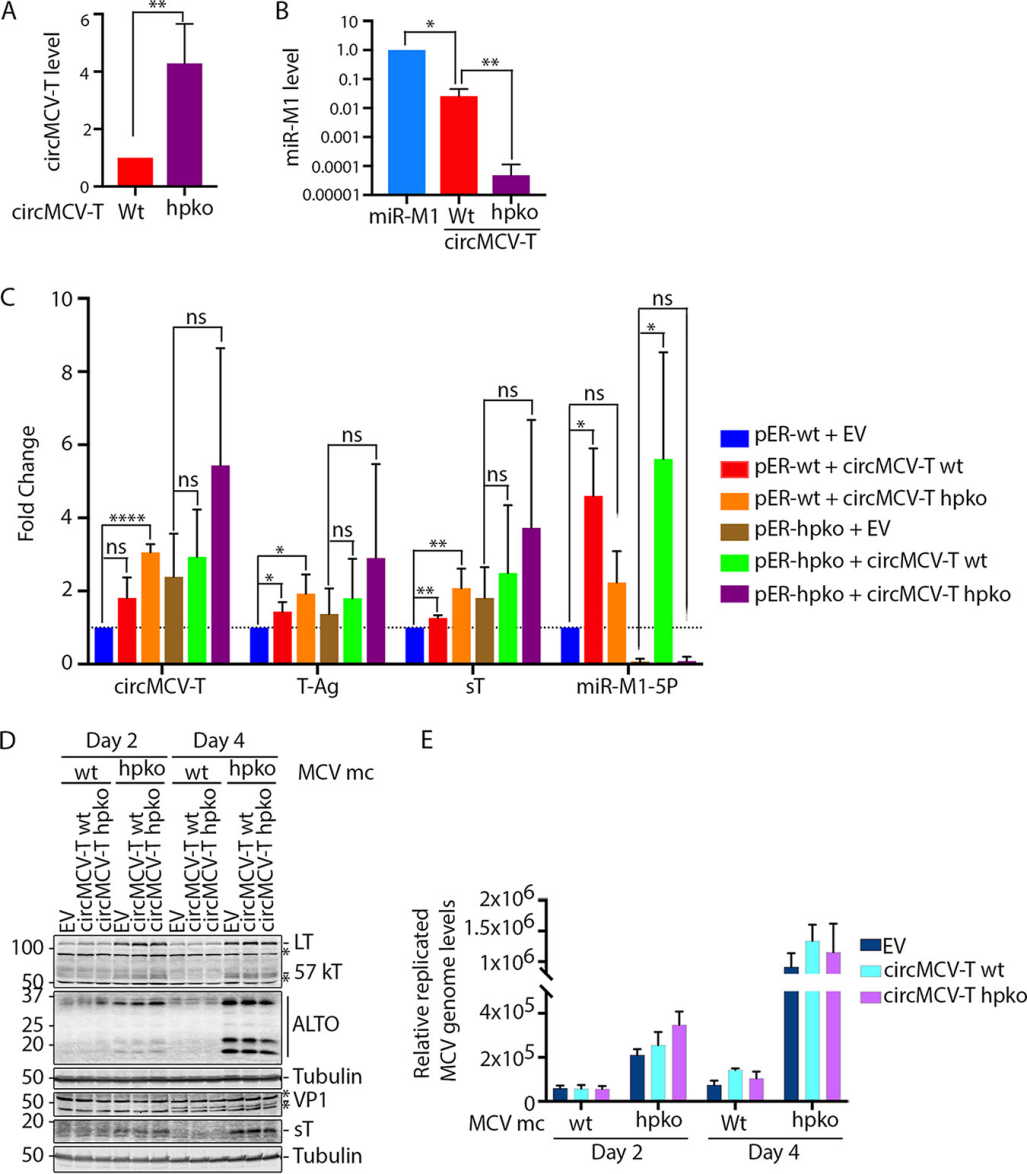
Effect of circMCV-T on MCV early transcript expression and viral replication. (A and B) 293 cells were transfected with the MCV miR-M1 or the circMCV-T wt or hpko mutant expression vector for 48 h, and RT-qPCR was performed for circMCV-T (A) and miR-M1-5P (B). (C) 293 cells were transfected with pCMV-ER wt or hpko constructs together with the circMCV-T wt or hpko mutant expression vector or an empty vector (EV) control for 48 h. RT-qPCR was performed for circMCV-T and for linear transcripts sT and LT as well as miR-M1-5P. *C_T_* values were normalized to RNase P or GAPDH *C_T_* values, and samples were normalized to EV-transfected cells for both the wt and hpko MCV ER construct to calculate fold change. Bars and error bars in panel C indicate means ± SD from three independent experiments. Statistical analysis was performed using an unpaired *t* test in panel A, an unpaired *t* test with Welch’s correction in panel B, and ordinary one-way analysis of variance (ANOVA) in panel C. Significance levels: *, *P* < 0.05; **, *P* < 0.01; ***, *P* < 0.001; ****, *P* < 0.0001; ns, not significant. (D and E) 293 cells were cotransfected with a wt or hpko mutant MCV-HF mc construct together with a circMCV-T wt or hpko mutant expression vector or an EV control. Cells were collected after 2 and 4 days of transfection. (D) Western blot analysis of MCV-encoded proteins (LT, 57kT, ALTO, sT, and VP1; α-tubulin was used as an internal control). (E) Quantification of the replicated (DpnI-resistant) MCV genome by qPCR analysis. Genomic GAPDH was used to normalize *C_T_* values, and relative levels of the MCV genome were calculated according to the ΔΔ*C_T_* method. Bars and error bars represent means ± SD from 3 replicates. Results are representative of three independent experiments.

We have shown that, as with the linear T-Ag transcripts, circMCV-T can be targeted by miR-M1. This predicts that circMCV-T plays a role in aiding viral replication by competing for miR-M1 binding with T-Ag linear transcripts early in the viral life cycle. To test this hypothesis, the wt MCV ER or that harboring the hpko mutation was cotransfected with wt or hpko mutant pLaccase-circMCV-T or an empty vector control, followed by RT-qPCR assessment of circMCV-T, miR-M1-5P, and linear transcripts. In addition to circMCV-T, the wt pLaccase-circMCV-T expression construct makes miR-M1 from the antisense strand (Fig. 5A and B). Figure 5C and Fig. S2 show that, as with the previous experiment (Fig. 4B), circMCV-T is expressed from both the wt and hpko mutant MCV ERs. Coexpression of the wt circMCV-T vector further increased circMCV-T levels both in the wild-type and hpko MCV ER constructs, while an hpko mutant pLaccase-circMCV-T further amplified the increase in circMCV-T expression levels. This increase in circMCV-T expression was accompanied by an increase in the level of sT and LT transcripts in both the wt and hpko mutant MCV ER constructs. As expected, miR-M1 levels were diminished in cells transfected with hpko mutant constructs. While the increase in miR-M1 expression from the MCV ER construct cotransfected with the wt circMCV-T is expected due to additive miRNA expression from a previously described promoter (2) present in the pLaccase circMCV-T construct (Fig. 5B), we have also observed a marked increase in miR-M1 expression from the MCV ER upon cotransfection of hpko mutant pLaccase-circMCV-T. In addition, there was also an increase in miR-M1 expression from the wt pLaccase-circMCV-T construct when cotransfected with the hpko mutant MCV ER construct (Fig. 5C). This may suggest regulation of the miR-M1 promoter by the increased level of T-Ag proteins from the hpko mutant MCV ER. To investigate whether the increase in linear T-Ag transcripts correlates with T-Ag protein expression and viral replication, the MCV-HF mc genome (MCV mc) with either the wt or hpko mutant miR-M1 sequence was cotransfected with wt or hpko mutant pLaccase-circMCV-T, and viral replication was assessed by Western blotting of MCV proteins and qPCR analysis of replicated viral genomes. Figure 5D shows that the overexpression of wt or hpko mutant circMCV-T does not result in a marked change in any of the MCV-encoded proteins from either the wt or the hpko mutant MCV mc genome. Further analysis of the level of replicated (DpnI-resistant) MCV genomes in these cells revealed only a slight increase at day 2 and day 4 posttransfection for hpko and wt MCV mc-transfected cells, respectively (Fig. 5E). However, the hpko mutation in the viral genome increased both viral protein expression and genome replication compared to levels in the wt MCV-HF mc genome (Fig. 5D and E).

10.1128/mBio.03059-20.2 FIG S2 Effect of circMCV-T on MCV early transcript expression. 293 cells were transfected with pCMV-ER wt or hpko constructs together with the circMCV-T wt or hpko mutant expression vector or an empty vector (EV) control for 48 h. RT-qPCR was performed for circMCV-T and linear transcripts sT and LT. *C_T_* values were used to calculate copy numbers per microgram of total RNA of each transcript shown from a standard curve. Actual copy numbers for each transcript are shown at the bottom of the graph. The bar graphs shown are representative of three independent experiments. Download FIG S2, TIF file, 1.0 MB. Copyright © 2020 Abere et al. 2020 Abere et al. This content is distributed under the terms of the Creative Commons Attribution 4.0 International license.

### Identification of RatPyV2-encoded circRNAs by RNase R+ RNA sequencing.

We performed RNase R^+^ sequencing on parotid gland RNA of a naturally infected SCID rat supporting active rat polyomavirus 2 (RatPyV2) viral replication (54). This model provides the advantage of an episomally replicating viral system under natural infection conditions in comparison to the MCV artificial transfection system in 293 cells. We identified 4 circRNAs, circRatPyV2_4112_4468 (the region homologous to circMCV-T), circRatPyV2_1653_1998, circRatPyV2_850_4468, and circRatPyV2_786_1266, from the ER using the RatPyV2 genome (NCBI accession no. KX574453.1) as a reference (Fig. S3 and Table S4). There were also two (RatPyV2_561_3856 and RatPyV2_561_3308) BSJ reads from the RatPyV2 LR; however, although the RatPyV2 LR transcript is not fully annotated, these reads are likely to also result from leader-to-leader forward splicing events, in like fashion to the MCV BSJ-simulating reads from the MCV late region, instead of from a bona fide backsplicing process. As with MCV, the contribution of BSJ reads from RatPyV2 to the total RNase R resistance reads in this system is also very low (<0.001 RPM). Nevertheless, the most abundant putative RatPyV2 circRNA from the ER, circRatPyV2_4112_4468, uses backsplicing sites (splice acceptor AG/GT and splice donor/GTAAGT) homologous to the circMCV-T (Table S2), suggesting a common mechanism of posttranscriptional RNA processing between the two polyomaviruses.

10.1128/mBio.03059-20.3 FIG S3 Identification of RatPyV2-encoded circRNAs by RNase R+ RNA sequencing. Schematic representation of the RatPyV2 genome organization (top) and RNase R+ RNA sequence coverage of RatPyV2 in the parotid gland of an infected X-SCID rat (bottom). Sequence reads from the forward strand (green) and reverse strand (magenta) are shown. Download FIG S3, TIF file, 1.1 MB. Copyright © 2020 Abere et al. 2020 Abere et al. This content is distributed under the terms of the Creative Commons Attribution 4.0 International license.

10.1128/mBio.03059-20.7 TABLE S4 Sequenced BSJs from RatPyV2. Download Table S4, PDF file, 0.04 MB. Copyright © 2020 Abere et al. 2020 Abere et al. This content is distributed under the terms of the Creative Commons Attribution 4.0 International license.

## DISCUSSION

circRNAs are a new class of closed circular transcripts whose biological relevance is becoming increasingly appreciated. Circular RNA transcripts have recently been recognized to be an important component of the transcriptome profiles of the human oncogenic DNA viruses EBV, KSHV, and HPV (41–43, 45). In this study, we show that another human DNA tumor virus, MCV, encodes circRNAs from its early region (ER). Further, transcriptome analysis of RatPyV2 identified BSJs in virus-infected rat tissues harboring an actively replicating episomal RatPyV2 genome (54), supporting the notion that expression of circRNA can reasonably be generalized to the family of polyomaviruses, not just human polyomaviruses or oncogenic polyomaviruses.

MCV circRNA BSJs were detected by RNase R sequencing only from cells supporting active viral replication. The absence of circMCV-T detection by RNase R sequencing from MCC-derived cell lines, which do not support active viral replication, can be attributable in part to the low level of viral transcripts expressed in these cells as well as viral genome integration, which may disrupt continuous ER RNA transcripts needed as the templates for backsplicing. Follow-up studies using more sensitive and directed PCR detection methods on a panel of MCC cell lines, all with nonreplicating integrated viral genomes and expressing tumor-specific signature T antigen truncations, show that some (CVG-1 and WaGa) do express circRNAs. Nevertheless, even with the increased sensitivity of direct PCR detection, several MCV-infected MCC cell lines (MKL-1, MKL-2, and MS-1) do not (Fig. 2C). The circMCV-T BSJ was detected by RT-PCR in two out of three patient MCC tumors tested. The third clinical sample did not have intact RNA after extraction. Expanded testing of fresh tumor samples will clarify whether circRNAs are expressed in all tumors or in only a subset, similar to what is seen with MCC-derived cell lines.

The most abundant MCV circRNA, circMCV-T, incorporates the entire exon II from early region pre-mRNA through backsplicing. Consistently, we find that circMCV-T expression patterns mirror 57kT transcript expression in MCC-derived cell lines. Both circMCV-T and the 57kT transcripts were detected in CVG-1 and WaGa but not in MKL-1, MKL-2, or MS-1 cells (Fig. 2C). Like other MCC-derived cell lines and primary tumors, CVG-1 expresses a truncated LT protein. In the case of CVG-1, the truncated product is due to a premature stop codon encoded by a single A-T mutation at nt 1617 (52). This tumor-associated mutation does not change the splicing motif at either the donor (at nt 1622) or the acceptor (at nt 861) site required for circMCV-T backsplicing. WaGa cells, derived from ascites fluid (51, 55), similarly express a truncated LT protein due to a single C-T mutation at nt 1461, which also is not predicted to affect circMCV-T formation. Additionally, both of the circMCV-T-positive cell lines WaGa and CVG-1 contain a relatively high number of integrated viral genome copies per cell, in contrast to MKL-2 and MS-1 cells, which do not express circMCV-T (52, 55). This may explain the increased circMCV-T and 57kT levels detected in these cell lines (Fig. 2C). On the other hand, MKL-1 cells, which display integrated viral genome copy numbers comparable to those of CVG-1 cells (52), also did not express detectable levels of circMCV-T (Fig. 2C); however, a 46-nt deletion between nt 1612 and 1657 abrogates the circMCV-T backsplice donor site at position 1622 in these cells. Using a single primer pair that can amplify and distinguish between both linear LT and 57kT transcripts in the same PCR, the full-length LT mRNA product is detected only in MCV-HF recircularized-genome-transfected cells and not in either the MCC-derived cell lines or primary tumor samples (Fig. 2C and D). In contrast, the sT transcript is detected at a relatively consistent level across cell lines and primary tumors tested. This suggests that transcripts that give rise to sT proteins may be differentially regulated from other T antigen transcripts.

Some circRNAs have been shown to code for a protein product through an IRES or m^6^A-mediated cap-independent translation initiation (39, 40, 45, 56). However, we did not find either endogenous or overexpressed circMCV-T to be significantly associated with polysomes, and thus it is unlikely to encode protein(s) (Fig. 3C and D). Although the circMCV-T sequence contains the start codon for the ALTO translation frame, there is no stop codon incorporated in the circle from the same reading frame (Fig. S1A). Western blot analysis, using antibodies that recognize the LT reading frame (CM2B4) or the ALTO reading frame (CM7B1) in the circMCV-T coding region, also did not detect a protein product from circMCV-T (Fig. S1B).

In contrast to most circRNAs, which are resistant to exonuclease digestion due to the absence of free RNA ends (22, 27), MCV-encoded circMCV-T is sensitive to RNase R treatment, suggestive of linearization. Nevertheless, RPAD shows the presence of true circular forms of circMCV-T, albeit at low abundances (Fig. 2E). We considered that this might be explained by the presence of MCV miRNA. The MCV genome in the same region that produces circMCV-T also elaborates an miRNA, miR-M1 from the antisense strand, which is processed into two mature miRNA seed sequences, miR-M1-5P and miR-M1-3P, that are 100% complementary not only to linear T-Ag transcripts but also to circMCV-T (2, 19). Perfect sequence complementary between an miRNA and its target sequence is predicted to result in RISC-mediated endonucleolytic cleavage of the target RNA. Recent studies (25, 57) have shown that such an interaction between miR-671 and a circRNA target CDR1-as results in Ago2-mediated cleavage and degradation of the circRNA. In support of this notion, MCV miR-M1 has been shown to target and cleave T-Ag transcripts to regulate MCV replication and promote episomal persistence (2, 19). Consistently with what occurred after miR-M1-mediated linearization of circMCV-T, exogenous expression of miR-M1 led to decreased circMCV-T levels accompanied by increased RNase R sensitivity (Fig. 4D and E), and hpko mutagenesis, which removes miR-M1 expression from the MCV ER, increased circMCV-T levels compared to those of a wt construct (Fig. 4B and 5C). miR-M1 has been shown to dominate the spectrum of miRNAs expressed in cells harboring replicating MCV episomes, in contrast to MCC-derived cells and primary tumors, which expresses miR-M1 only at very low levels (2, 58). This might explain the sparse amounts of viral circRNA BSJs detected from cells with replicating MCV genomes in our current study. The provocative directionality of the depletion of sequence coverage at the circRNA locus (Fig. 1B) may be attributable to the 3′-to-5′ exonuclease activity preference of RNase R. It is also possible that circMCV-T itself forms a hairpin structure that can be processed by Drosha in a fashion similar to that of the pre-miR-M1 RNA from the opposite strand, which would in the end lead to its RNase R sensitivity. Future experimentation using Drosha knockout cells may shed light on this.

Another intriguing consequence of circMCV-T and MCV miRNA interaction is the effect of circMCV-T on its targeting miRNA. Although miRNAs are usually stable due to protection by the Argonaut protein complex from exonucleolytic degradation, accumulating evidence suggests that extensive complementarity between a target RNA and an miRNA may lead to target RNA-directed miRNA degradation (TDMD) (57, 59–61). Since mature miR-M1 products can bind circMCV-T with 100% complementarity, this may suggest a mechanism that promotes MCV miR-M1 for depletion during active viral replication.

**FIG 6:**
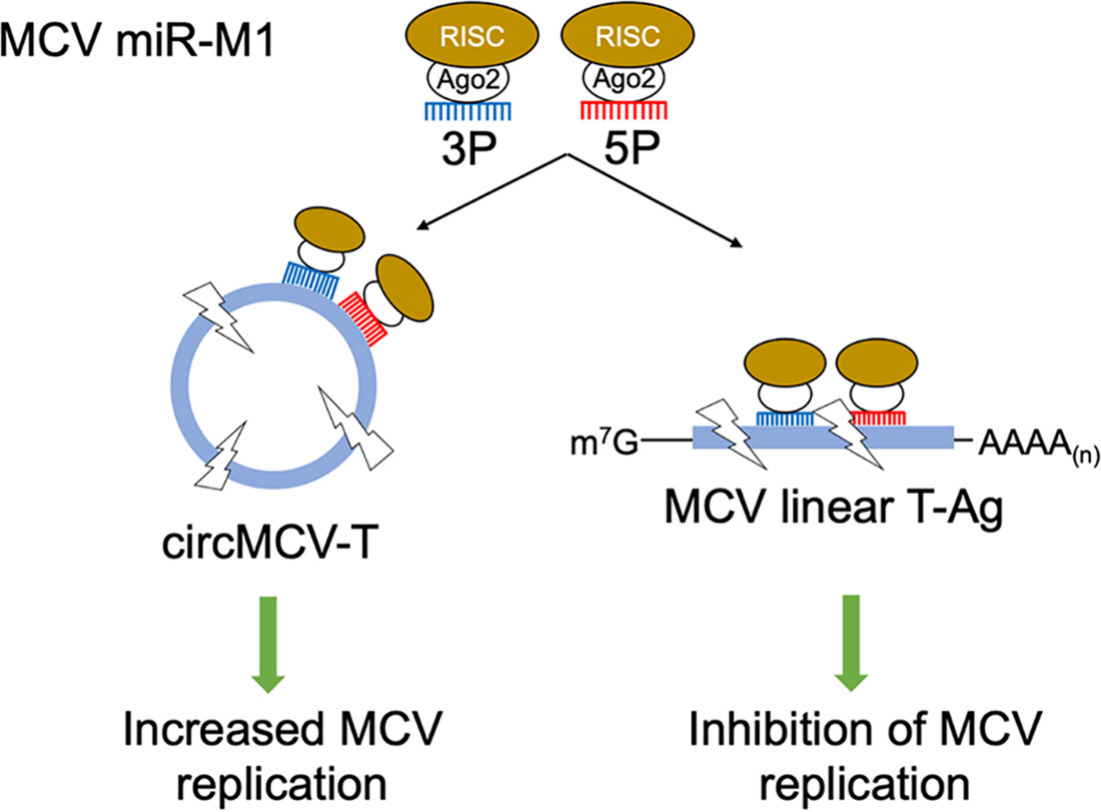
Working model for MCV circMCV-T regulation of MCV T-Ag expression. MCV miR-M1 is incorporated into the RISC complex, which will target both circMCV-T and linear T-Ag transcripts. If circMCV-T absorbs the miR-M1-loaded RISC complex, linear T-Ag transcripts will be stabilized and T-Ag expression will increase. In the absence of circMCV-T, linear T-Ag transcripts will be degraded by the miR-M1-induced RISC complex, which leads to inhibition of T-Ag expression and, therefore, MCV replication.

Expression of circMCV-T increased T-Ag (sT and LT) transcripts only to a modest level (Fig. 5C). This can be, in part, due to additional expression of miR-M1 from the antisense strand of the pLaccase-circMCV-T expression construct (Fig. 5B) and, thus, to further degradation of linear T-Ags canceling out the effect of circMCV-T expression. An additional mechanism for a circMCV-T-mediated increase in linear T-Ag transcripts independent of circMCV-T’s role as a decoy to miR-M1 is shown by the increase in the levels of these transcripts in cells cotransfected with hpko mutant pLaccase-circMCV-T, which lacks miR-M1 binding sites (Fig. 5A). Consistently, coexpression of the hpko mutant of the MCV ER together with an hpko-circMCV-T resulted in the highest level of T-Ag transcripts, although this increase is not statistically significant. Regardless, exogenous expression of circMCV-T promotes MCV replication to only a limited extent (Fig. 5D and E), while abrogating miR-M1 expression from the viral genome alone substantially increased T-Ag protein expression and viral replication, consistent with the results of previous reports (2). This reciprocal interaction between two different classes of viral RNAs reveals an entirely new level of complexity in the interplay between viral gene products. In the presence of circMCV-T, miR-M1 is functionally sequestered from linear T-Ag transcripts that code for replication proteins, and therefore, MCV replication is favored; at low levels of circMCV-T, miR-M1 binds and degrades linear T-Ag transcripts and thus suppresses active viral replication to promote persistent infection. Finally, competitive splicing may also be an attractive concept for circMCV-T function. Because circMCV-T uses the same splice sites as canonical linear splicing events for LT-Ag and 57kT, its production may come at the expense of these linear transcripts. This may be a mechanism that can shift the balance of T-Ag expression toward sT. In this scenario, the presence of noncoding circMCV-T molecules that present miR-M1 sites may be an additional factor that protects sT Ag transcripts. circMCV-T may be part of a program that includes sT transcript regulation to enhance lytic virus replication; however, miR-M1 expression suppresses lytic replication to establish MCV latency. Our findings suggest that MCV-encoded circRNAs represent another layer of regulation to fine-tune MCV replication through a balanced titration of three classes of MCV transcripts: circMCV-T, miR-M1, and mRNAs (Fig. 6).

## MATERIALS AND METHODS

### Tumor samples and cell lines.

Three freshly frozen tumor tissue specimens (MCC-1, MCC-2, and MCC-3) from patients with MCV-positive MCC were obtained from the Cooperative Human Tissue Network (CHTN). RatPyV2-positive parotid salivary gland tissue was obtained from a rat with X-linked severe combined immunodeficiency (X-SCID) (54) according to the University of Pittsburgh animal care and use committee guidelines (protocol number 16048182). MCV-positive MCC tumor-derived cell lines WaGa (51), MKL-1 (62), MKL-2 (63), CVG-1 (52), and MS-1 (64) were maintained in Roswell Park Memorial Institute (RPMI) 1640 medium (Cellgro) supplemented with 10% fetal bovine serum (FBS; VWR Seradigm). 293 cells (ATCC CLR 1573) were maintained in Dulbecco's modified Eagle medium (DMEM; Corning) supplemented with 10% FBS. Transfections were performed using either Lipofectamine 2000 (Life Technologies) or FuGENE 6 (Promega) by following the manufacturers’ protocols.

### Plasmids and constructs.

Construction of the consensus pJMCV-HF plasmid for the production of the circular, wild-type MCV-HF genome is detailed in reference 7. Plasmids pCDNA3.1-ER-wt, pCDNA3.1-ER-hpko, pCDNA3.1GFP-MCV-miR-M1-wt 300, and pCDNA3.1GFP-MCV-miR-M1- hpko 300 have also been previously described (2). For ectopic circRNA expression, we made use of the pcDNA3.1 (+) Laccase2 MCS exon vector, which models a *Drosophila* Laccase 2 miniature intron incorporating flanking inverted repeats and splice sites to facilitate circularization. The pcDNA3.1 (+) Laccase2 MCS exon vector was a gift from Jeremy Wilusz (catalog [cat.] no. 69893; Addgene) (65). The pLaccase-circMCV-T construct was generated by amplification of the circMCV-T coding region from the MCV-HF genome (GenBank accession no. JF813003) using primers circ_MCV_LT_PacI_F and circ_MCV_LT_SacII_R, with subsequent cloning into the pcDNA3.1 (+) Laccase2 MCS exon vector using PacI and SacII restriction sites. pLaccase circMCV-T-hpko was similarly made using the same primer pairs but with pCMV-ER-hpko as the template. The pMC.BESPX-MCV-HF plasmid used for production of the MCV-HF mini-circle was synthesized (GenScript). The MCV-HF genome was first synthesized with SmaI and BstEII restriction sites inserted into the MCV-HF genomic sequence between the ER and LR (between nt 3146 and 3147). The mini-circle plasmid vector pMC.BESPX (cat. no. MN100A-1; System Biosciences) (66–68), a kind gift from Mart Ustav (University of Tartu, Tartu, Estonia) and Alison McBride (National Institute of Allergy and Infectious Diseases, Bethesda, MD), was then cloned into the synthesized MCV-HF genome using restriction sites SmaI and BstEII to generate pMC.BESPX-MCV-HF. To make the hairpin mutant pMC.BESPX-MCV-HF-hpko mini-circle plasmid, a 1.6-kb fragment (nt 1152 to 2827) containing the MCV-miR-M1 region from the pMC.BESPX-MCV-HF plasmid was swapped with a similar fragment from the pCMV-ER-hpko plasmid using EcoRI and BamHI restriction sites. All constructs were validated and confirmed by sequencing. The list and sources of all constructs are shown in Table 1.

**TABLE 1:** List and description of plasmids

Construct	Function^*b*^	Parental vector	CM plasmid no.	CM plasmid name
MCV-HF	Amplifies the MCV-HF genome in bacteria for MCV genome recircularization by religation after enzyme digestion that releases the MCV-HF genome from the bacterial backbone	pJ	3147	MCV-HF
MCV-wt-mc	Amplifies the MCV-HF genome in bacteria for MCV genome recircularization by recombination	pMC.BESPX	4587	pMC.BESPX-MCV-HF
MCV-hpko-mc	Amplifies MCV-HF-hpko, which diminishes MCV miR-M1, in bacteria for MCV genome recircularization by recombination	pMC.BESPX	4610	pMC.BESPX-MCV-HF-hpko
pER-wt^*a*^	Expresses all early gene products, including MCV miR-M1	pCDNA3.1	4427	pCMV2b-ER(pCMV-ER)
pER-hpko^*a*^	Expresses all early gene products except for MCV miR-M1	pCDNA3.1	4498	pCMV2b-ER-hpko
miR-M1 wt^*a*^	Expresses MCV miR-M1 and GFP	pCDNA3.1	4429	pCDNA3.1GFP-MCV-miRM1 300 (pCMV-miRNA)
miR-M1 hpko^*a*^	Expresses GFP only; negative control for MCV miR-M1 expression	pCDNA3.1	4499	pCDNA3.1-GFP-MCV-miRM1 hpko 300
circMCV-T wt	Expresses circMCV-T; this plasmid also has all elements to express MCV miR-M1 in the negative strand	pcDNA3.1 (+) Laccase2 MCS (Addgene no. 69893)	4448	pcDNA3.1 (+) Laccase-circMCV-LT
circMCV-T hpko	Expresses circMCV-T that cannot produce or interact with MCV miR-M1	pcDNA3.1 (+) Laccase2 MCS (Addgene no. 69893)	4643	pcDNA3.1 (+) Laccase-circMCV-LT-HPKO.SNPFIXED
EV	The backbone of pLaccase-circMCV-T-wt and pLaccase-circMCV-T-hpko, a negative control for circMCV-T expression	pcDNA3.1 (+) Laccase2 MCS (Addgene no. 69893)	4444	pcDNA3-Laccase-MCS (AA mut)

a These constructs were generated in the laboratories of Nicole Fischer and Adam Grundhoff (see reference 2).

b GFP, green fluorescent protein.

### MCV-HF recircularization and mini-circle production.

Release and recircularization of the MCV-HF genome from pJ-MCV-HF was previously described (7). To produce MCV genomes using the mini-circle technology (66–68), the pMC.BESPX-MCV-HF or pMC.BESPX-MCV-HF-hpko plasmid was transformed into ZYCY10P3S2T (cat. no. MN900A-1; System Biosciences) competent cells, a kind gift from Mart Ustav (University of Tartu, Tartu, Estonia) and Alison McBride (National Institute of Allergy and Infectious Diseases, Bethesda, MD). Single colonies were inoculated into 5 ml of LB with 50 μg/ml kanamycin and grown at 37°C for approximately 8 h. One hundred microliters of the starter culture was added into 400 ml of Terrific broth and cultured overnight at 37°C. The next morning, when an optical density at 600 nm (OD_600_) of between 4 and 5 was reached, an equal volume of induction mix (40 mM NaOH and 0.02% l-arabinose in a total of 400 ml LB) was added and the culture was grown for an additional 5 h at 32°C to induce mini-circle production. DNA was extracted with Maxiprep (Macherey-Nagel), and recombination efficiency was confirmed by restriction digestion. The resulting MCV-HF mini-circle genome retains a 39-bp scar of bacterial plasmid sequence (GCCCCAACTGGGGTAACCTTTGGGCTCCCCGGGCGCGAC) between nucleotides 3146 and 3147.

### RNA isolation and circRNA sequencing.

Total RNA was extracted from 293 cells transfected with the MCV-HF recircularized genome (5 days posttransfection), MCC cell lines (CVG-1 and MS-1), and RatPyV2-positive parotid gland tissue using TRIzol reagent (Invitrogen) and further processed with a Turbo DNase kit (cat. no. AM190; Invitrogen) according to the manufacturer’s instructions. RNA quality was confirmed by Agilent TapeStation (Children’s Hospital of Pittsburgh of UPMC, sequencing core facility) and with an Agilent 2100 Bioanalyzer (CD Genomics). RNase R-treated samples were used for library preparation and subsequent circRNA sequencing using the Illumina HiSeq platform in PE150 sequencing mode (CD Genomics).

### Bioinformatic analysis.

Raw FastQ files were trimmed with Trim Galore (http://www.bioinformatics.babraham.ac.uk/projects/trim_galore/) using the following parameters: q 25, e 0.1, and length 50, and the quality control was performed with FastQC. circRNA prediction was conducted with CIRI2 (47; https://sourceforge.net/projects/ciri/files/CIRI2/) with the default settings. RNA sequencing (RNA-seq) reads were aligned to MCV-HF (NCBI accession no. JF813003) or RatPyV2 (accession no. KX574453) reference genomes using BWA mapper. CLC genomics workbench (Qiagen) was used to align RNA-seq reads to MCV-HF (GenBank accession no. JF813003) and RatPyV2 (accession no. KX574453) reference genomes and to visualize additional annotation tracks using the following parameters: mismatch cost of 10, insertion cost of 3, deletion cost of 3, length fraction of 0.5, and similarity fraction of 0.9.

### RNase R treatment and RPAD.

One microgram of DNase-digested total RNA was treated with 4 U of RNase R (NCBI accession no. RNR07250; Lucigen) in 1× RNase R reaction buffer at 37°C for 30 min in the presence of 8 U of RiboLock RNase inhibitor (cat. no. EO0381; Thermo Scientific); for untreated samples, nuclease-free water was added to the reaction mixture instead of RNase R. RNase R was inactivated by incubation at 65°C for 20 min. RPAD was performed as previously described (53). Briefly, 2 μg of RNase R-treated RNA was ethanol precipitated in the presence of sodium acetate and 20 μg of glycogen as a carrier, followed by polyadenylation using the E. coli PolyA Polymerase I (E-PAP) kit (AM1350; ThermoFisher) in the presence of RiboLock RNase inhibitor. Control RNA was processed in the same way without the E-PAP buffer. Polyadenylated RNA was then depleted using a Purist MAG kit (cat. no. AM1922; ThermoFisher). cDNA was then synthesized as described below.

### Semiquantitative and quantitative RT-PCR.

One microgram of DNase-treated total RNA in the presence or absence of RNase R treatment was reverse transcribed using random hexamers and the SuperScript IV first-strand synthesis system (cat. no. 18091; Invitrogen). Semiquantitative PCR for circRNA detection was performed using Q5 high-fidelity DNA polymerase (cat. no. M0491; New England BioLabs/NEB) and *Taq* DNA polymerase with ThermoPol buffer (cat. no. M0267; NEB) for linear RNA detection, according to the respective protocols from the manufacturers. See Table 2 for the list of PCR primers used in this study.

**TABLE 2:** PCR primers

Primer name	MCV genome position^*a*^	Sequence
circMCV-T_DP1F (DP1)	1514–1533	TACAAGCACTCCACCAAAGC
circMCV-T_DP1R1 (DP1R1)	926–946	TATTCGTATGCCTTCCCG
circMCV-T_DP1R2 (DP1R2)	1046–1067	GGACCCATACCCAGAGGAAGAG
circMCV-T_DP2F (DP2)	1409–1428	TGGTGAAGGAGGAGGATCTG
circMCV-T_DP2R (DP2)	1483–1503	GCTCTGCAAGCTCTGCTAGTT
circMCV-T_DP3F (DP3)	1079–1099	CCAGGCTTCAGACTCCCAGTC
circMCV-T_DP3R (DP3)	1057–1078	GACGCTGAGAAGGACCCATACC
circSMARCA5_F	NA	CTCCAAGATGGGCGAAAGT
circSMARCA5_R	NA	TTCTGATCCACAAGCCTCC
U6_F	NA	GTGCTCGCTTCGGCAGCACA
U6_R	NA	AAAATATGGAACGCTTCACGA
Linear sT_F	397–422	AAGCTCAGAAGTGACTTCTCTATGTT
Linear sT_R	613–632	TCTCCCCACGTCAGACAGTT
Linear LT + 57 kT_R (CP-R)	3052–3080	TTATTGAGAAAAAGTACCAGAATCTTGGG
Linear β-actin_F	NA	CACACTGTGCCCATCTATGAGG
Linear β-actin_R	NA	TCGAAGTCTAGGGCGACATAGC
Circ_MCV_LT_PacI F	NA	CGCTTAATTAATTTCCCATCTAGGTTGAC
Circ_MCV_LT_SacII R	NA	GATCCGCGGACTTACTGTTTTATTACT

a NA, not applicable.

To detect mcv-miR-M1 by quantitative stem-loop RT-PCR, 1 μg of total RNA without DNase and RNase R treatment was reverse transcribed using a TaqMan microRNA reverse transcription kit (cat. no. 4366597; ThermoFisher) with an mcv-miR-M1-specific stem-loop primer (SL_MCV-miR-M1) and a GAPDH-specific primer (GAPDH rev) for normalization as described before (2). Quantitative PCR (qPCR) of mcv-miR-M1, GAPDH, MCV circMCV-T, and linear transcripts was performed on input cDNA using TaqMan universal master mix II with UNG (cat. no. 44400; Applied Biosystems) in a QuantStudio 5 real-time PCR system (ThermoFisher). See Table 3 for a list of qPCR primers and probes. Threshold cycle (*C_T_*) values were used to calculate expression levels. *C_T_* values for mcv-miR-M1 were normalized to GAPDH, other transcripts were normalized to RNase P, and expression levels were calculated according to the ΔΔ*C_T_* method.

**TABLE 3:** qPCR primers and probes^*a*^

Primer/probe name	Sequence	
TaqMan probe circMCV-T	6FAM-AAAACAGTTGACGAGGCCCCTATATATGGG-QSY
circMCV-T_qPCR_F	ACTCCTGTTCCTACTGATTTTCC
circMCV-T_qPCR_R	TCCTCCTGATCTCCACCATTC
SL_MCV-miR-M1	GTCGTATCCAGTGCAGGGTCCGAGGTATTCGCACTGGATACGACTGTACC
TaqMan probe MCV-miR-M1	ABY-CGCACTGGATACGACTGTACC-QSY
MCV-miR-M1 FW	GCATCTGGAAGAATTTCTA
Universal rev	GTGCAGGGTCCGAGGT
TaqMan probe GAPDH	ABY-GTGGCGCTGAGTACGTCGTGGAGTC-QSY
GAPDH BSP FW	GGTCGGAGTCAACGGATTTG
GAPDH rev	ATGGTGGTGAAGACGCCAGT
GAPDH DNA fw	TGTGTCCCTCAATATGGTCCTGTC
TaqMan Probe sT	ABY-AGCTGTAAGTTGTCTCGCCAGCATTGT-QSY
linear sT_qPCR_F	GCTAGATTTTGCAGAGGTCCT
linear sT_qPCR_R	AAAACACTCTCCCCACGTCA
TaqMan probe T-Ag	ABY-TGGAATTGAACACCCTTTGGAGCA-QSY
linear T-Ag_qPCR_F	TGCTCCTAATTGTTATGGCAAC
linear T-Ag_qPCRR	AGCTTGTGGATATTTTGCTGGA
POP4 (RNase P)	VIC-MGB Hs00198357_m1 (cat. no. 4331182)

a 6FAM, 6-carboxyfluorescein; QSY, QuantStudioY; ABY, AppliedBiosystemsY; VIC-MGB, VIC-Minor Groove Binder.

### BaseScope RNA *in situ* hybridization.

To prepare the cell pellet array, 293 cells were transfected with a pLaccase-empty vector, pLaccase-circMCV-T, or pER. After 48 h, transfected 293 cells and the MCC cell line CVG-1 were harvested, and pellets were fixed in 10% neutral buffered formalin and processed with HistoGel (cat. no. HG-4000-012; Thermo Scientific) for routine histology. Five-micrometer sections of formalin-fixed paraffin-embedded cell pellets were then stained for the respective RNA species using BaseScope RNA *in situ* hybridization with the BaseScope Duplex detection reagent kit (cat. no. 323810; Advanced Cell Diagnostics [ACD]) using probes targeting circRNA BSJ or linear transcripts by following a detailed protocol described previously (69). In this study, probes with a red or blue signal were combined for hybridization (the POLR2A probe is combined with the PPIB probe; the MCV circMCV-T probe is combined with the linear T-Ag probe) and developed on the same slide sequentially. First, the red signal was amplified and developed as described previously (69). Subsequently, slides were treated with AMP10 for 15 min at 40°C, AMP11 for 30 min at room temperature, and AMP12 for 15 min at room temperature, with washing steps after each incubation period. The chromogenic signal was developed by incubating cells with BaseScope Duplex Green solution for 10 min at room temperature in the dark. Images were acquired using an Olympus AX70 microscope with a QImaging QIClick charge-coupled device (CCD) camera and Q-Capture Pro 7 software. circMCV-T is detected with a single 1zz probe, BA-V-MCPyV-gp3-circRNA-Junc-C2 (cat. no. 722851-C2; ACD), to its BSJ, while a single 2zz probe, BA-V-MCPyV-gp3-2zz-st (cat. no. 722841; ACD), was used to detect linear T-Ag RNA. Since the circMCV-T probe is diluted with the linear T-Ag probe (1:50 dilution), the circMCV-T signal (red) and linear T-Ag signal (blue) are detected at the same time in two different channels. The human housekeeping genes *PPIB* and *POLR2A* were used as a positive control, while a probe against the bacterial DapB gene was used as a negative control (BaseScope Duplex control probe Hs-1zz; ACD cat. no. 700101).

### Polysome fractionation.

293 cells were transfected with 5 μg of pLaccase-circMCV-T plasmid DNA in a 10-cm plate for 48 h or with 2 μg of MCV-HF-hpko mc in the presence or absence of 2 μg of the pLaccase-circMCV-T-hpko plasmid in a well of a 6-well plate for 4 days posttransfection. CVG-1 cells (2 × 10^7^) were treated with cycloheximide (CHX; 100 μg/ml) and incubated for 15 min at 37°C in 5% CO_2_. Cells were then collected by centrifugation at 200 × *g* for 3 min, washed with cold PBS containing 100 μg/ml CHX, and pelleted by centrifugation at 200 × *g* for 3 min. One milliliter of lysis buffer (100 mM KCl, 5 mM MgCl_2_, 10 mM HEPES [pH 7.2 to 7.4], 0.5% NP-40, 100 μg/ml CHX, 1.5 mM dithiothreitol [DTT], 200 U/ml RiboLock RNase inhibitor) was added to the cell pellet and lysed at 4°C for 10 min. Cell lysates were centrifuged at maximum speed (17,000 × *g*) for 10 min at 4°C, the supernatant was laid over a sucrose gradient (1.2 ml 50%, 0.9 ml 40%, 0.9 ml 30%, 0.8 ml 20%, and 0.7 ml 10% sucrose, bottom to top) in ultracentrifuge tubes (cat. no. 344057; Beckman) and centrifuged at 35,000 RPM for 3 h at 4°C using an AH-650 ultracentrifuge rotor (Sorvall) and a Discovery 90SE ultracentrifuge (Sorvall). Samples were then subjected to fractionation on an ISCO density gradient fractionation system (Teledyne Technologies) using the following pump program: collection in microtubes; last tube, 15 (15 fractions); fraction by drops, 20 drops (300 μl); flow delay, 0 s; event, 25% pump output; event time, 0 s; and chart speed, 150 cm/h. Eight hundred microliters of TRIzol LS reagent (cat. no. 10296010; Invitrogen) was then added to each fraction, and RNA was extracted according to the manufacturer’s protocol. A random hexamer was used for cDNA synthesis, and qPCR was performed for circMCV-T and GAPDH using the TaqMan chemistry as described above.

### Immunoblotting.

Transfected 293 cells were collected in 1% SDS buffer (1% SDS, 10 mM Tris-HCl, pH 8.0, 1 mM EDTA, pH 8.0) and sonicated on ice four times for 5 s each time, and 100 μg of protein was used for SDS-PAGE. Protein was transferred to nitrocellulose membranes and incubated with a 1:500 dilution of CM2B4 (MCV LT-Ag and 57 kT antibody), a 1:10 dilution of CM7B1 (MCV ALTO antibody), a 1:500 dilution of CM9B2 (MCV VP1 antibody), and a 1:1,000 dilution of mouse α-tubulin antibody (12G10; DSHB). A 1:10,000 dilution of IRD800 anti-mouse antibody (cat. no. 926-32210; Rockland) was used as the fluorescent secondary antibody. Images were acquired on a ChemiDoc imaging system (Bio-Rad).

### MCV replication assay.

293 cells transfected with either a wt or an hpko mutant MCV-HF mini-circle genome were collected 2 and 4 days posttransfection using DNA lysis buffer (0.1 M NaCl, 10 mM Tris·HCl, pH 8.0, 25 mM EDTA, pH 8.0, 0.5% SDS, 200 μg/ml proteinase K) and incubated at 45°C overnight. DNA was extracted using standard phenol-chloroform (cat. no. P3803; Sigma) and precipitated with sodium acetate and ethanol. DNA was treated with BamHI and DpnI (New England Biolabs) overnight. Five nanograms of treated DNA was used for qPCR. Viral genomes were quantified with primers (Genomic VP1_F and Genomic VP1_R) spanning three DpnI restriction sites and normalized to GAPDH (primers GAPDH DNA fw and GAPDH-rev).

### Data availability.

RNase R+ sequencing data are deposited in the Gene Expression Omnibus (GEO) repository (https://www.ncbi.nlm.nih.gov/geo) under accession number GSE162627.
