# Supplementary material for: Merkel Cell Polyomavirus Encodes Circular RNAs (circRNAs) Enabling a Dynamic circRNA/microRNA/mRNA Regulatory Network
Source: mBio. 2020 Dec 15;11(6):e03059-20. doi: 10.1128/mBio.03059-20 (PMC7773998; doi:10.1128/mBio.03059-20)
Supplement: TABLE S1 [file mBio.03059-20-st001.pdf]

TABLE S1. Summary of RNaseR+ RNAseq reads obtained from MCC cell lines, MCV-HF transfected 293 cells, and RatPyV2 infected rat parotid gland.

	# total reads	# unmapped	# mapped	%mapped reads
**CVG**	85,674,624	85,673,165	1,459	0.0017
**MS1**	80,138,038	80,137,423	615	0.0008
**293-MCV-HF**	77,848,404	77,462,185	386,219	0.4961
**RatPyV2-parotid**	82,621,490	81,875,309	746,181	0.9031
