# Supplementary material for: Merkel Cell Polyomavirus Encodes Circular RNAs (circRNAs) Enabling a Dynamic circRNA/microRNA/mRNA Regulatory Network
Source: mBio. 2020 Dec 15;11(6):e03059-20. doi: 10.1128/mBio.03059-20 (PMC7773998; doi:10.1128/mBio.03059-20)
Supplement: TABLE S2 [file mBio.03059-20-st002.pdf]

TABLE S2: Sequenced BSJs from MCV-HF

MCV BSJ Start (nucleotide number)	MCV BSJ End (nucleotide number)	Strand	BSJ reads	RPM
861	1622	+	53	0.014
861	2955	+	9	0.002
861	3337	+	3	0.001
3271	3913	+	6	0.002
		Subtotal Forward	71	0.019
1142	5308	-	92	0.024
1142	5119	-	25	0.006
1142	4642	-	4	0.001
		Subtotal Reverse	121	0.031

MCV-HF= re-circularized genome transfected in to 293 cells, GenBank ID JF813003)
