# Supplementary material for: Merkel Cell Polyomavirus Encodes Circular RNAs (circRNAs) Enabling a Dynamic circRNA/microRNA/mRNA Regulatory Network
Source: mBio. 2020 Dec 15;11(6):e03059-20. doi: 10.1128/mBio.03059-20 (PMC7773998; doi:10.1128/mBio.03059-20)
Supplement: TABLE S3 [file mBio.03059-20-st003.pdf]

TABLE S3: circMCV-T BaseScope *IH* detection in CVG-1 cells

	Total	Linear T-Ag Positive cells	circMCV-T Positive cells
Number of cells	402	343	19
%	100	85.3	4.73
