# Supplementary figures and images for: Merkel Cell Polyomavirus Encodes Circular RNAs (circRNAs) Enabling a Dynamic circRNA/microRNA/mRNA Regulatory Network
Source: mBio. 2020 Dec 15;11(6):e03059-20. doi: 10.1128/mBio.03059-20 (PMC7773998; doi:10.1128/mBio.03059-20)

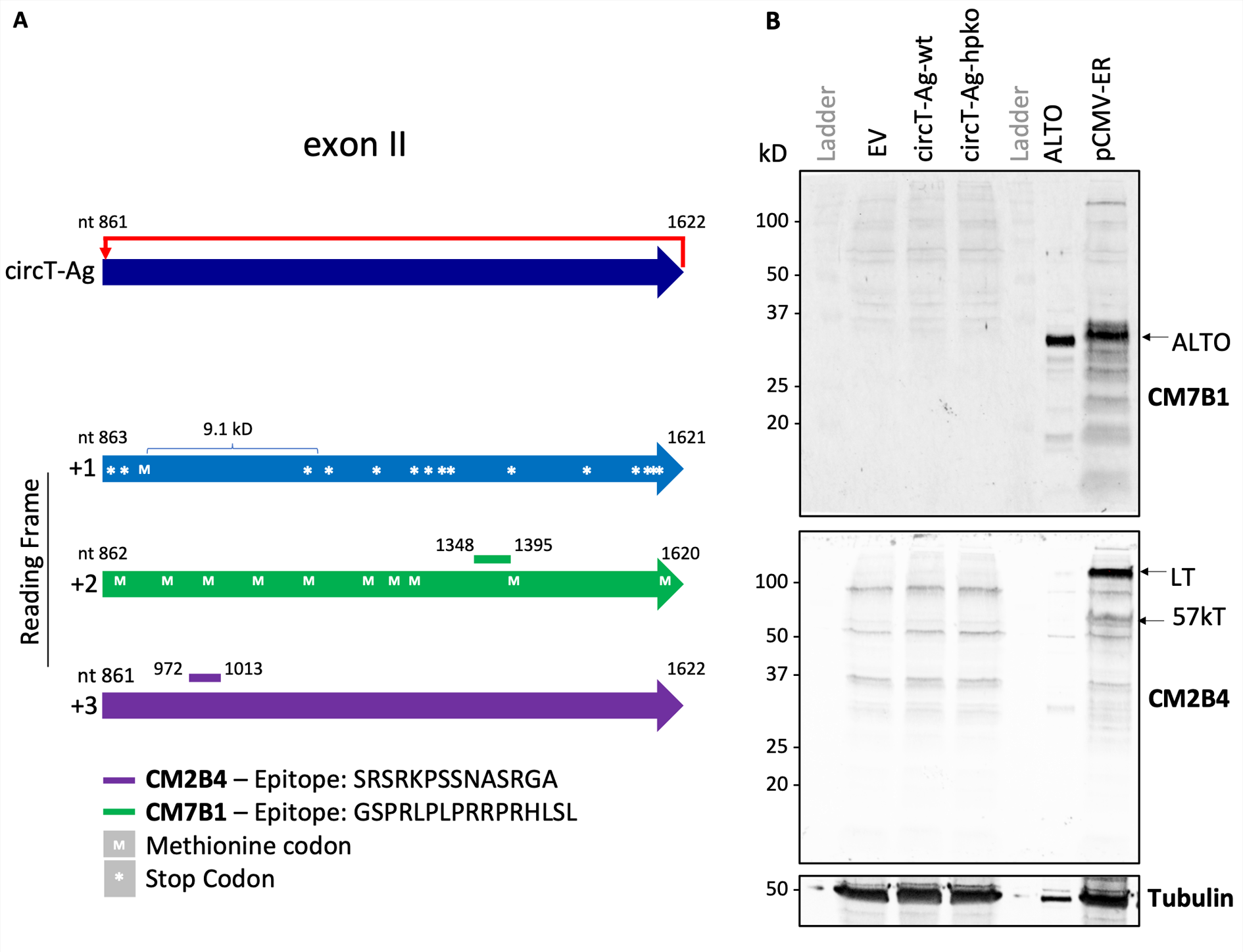

Supplement: FIG S1 [file mBio.03059-20-sf001.tif]

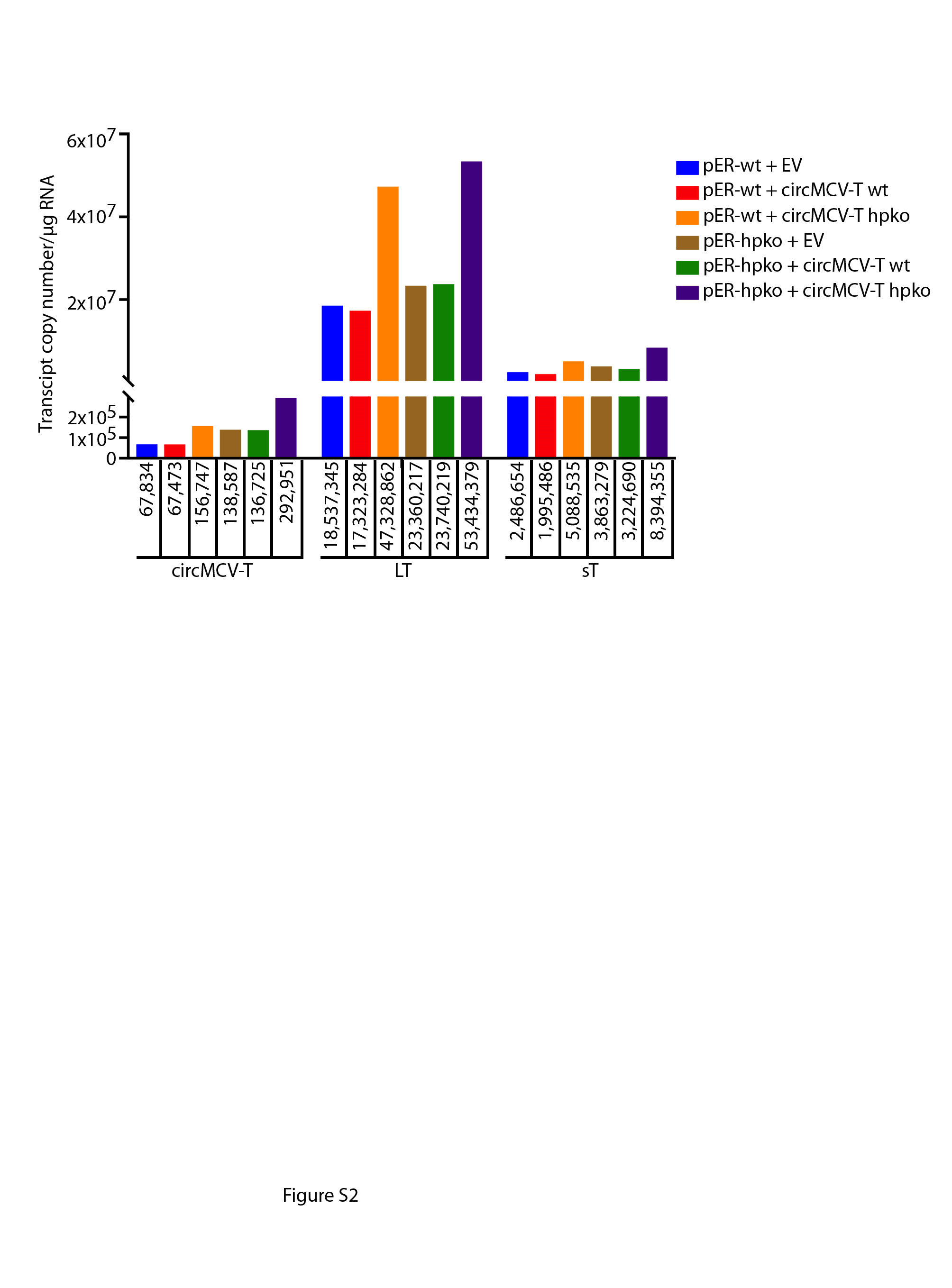

Supplement: FIG S2 [file mBio.03059-20-sf002.tif]

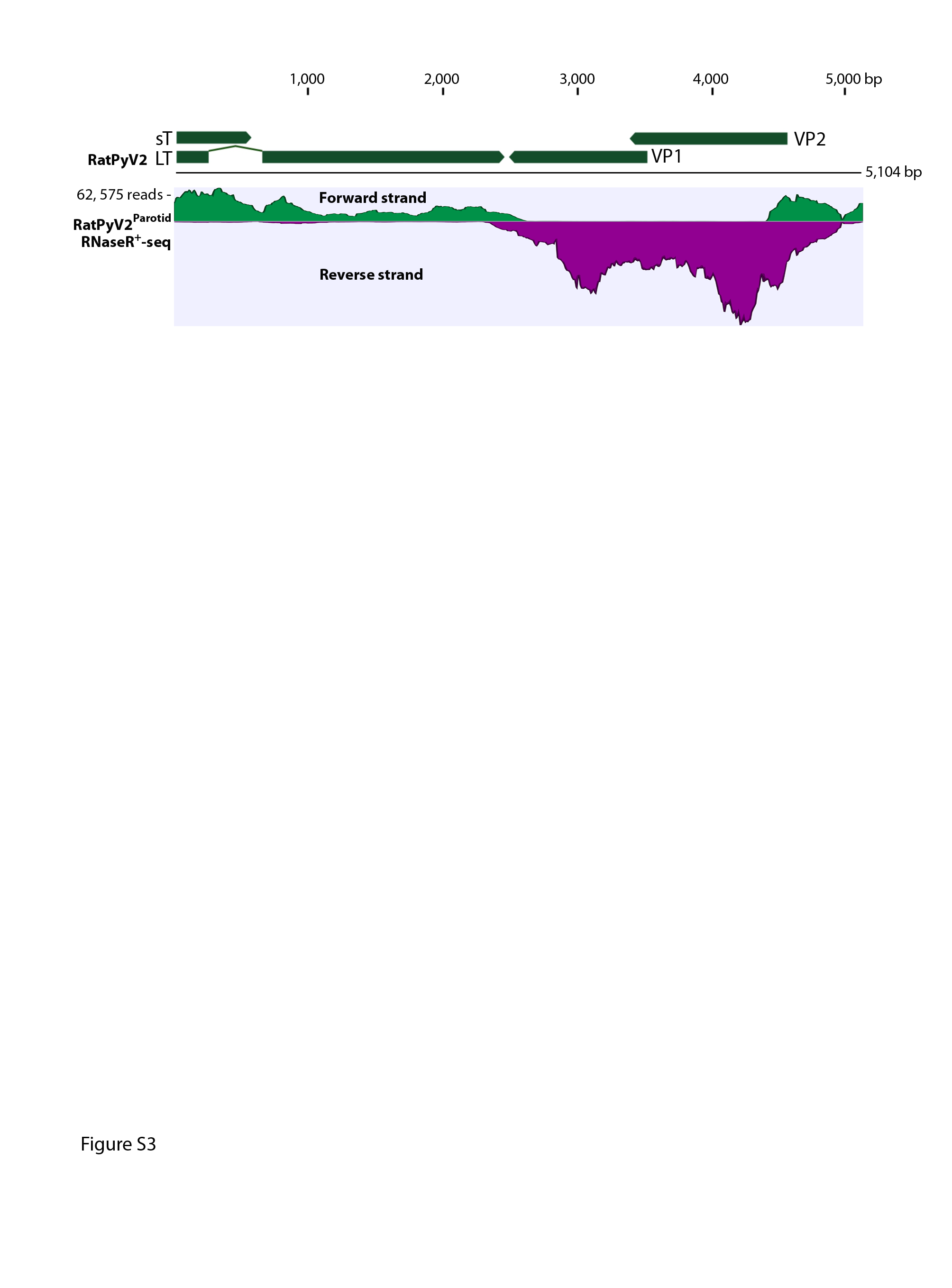

Supplement: FIG S3 [file mBio.03059-20-sf003.tif]
