# Supplementary material for: Merkel Cell Polyomavirus Encodes Circular RNAs (circRNAs) Enabling a Dynamic circRNA/microRNA/mRNA Regulatory Network
Source: mBio. 2020 Dec 15;11(6):e03059-20. doi: 10.1128/mBio.03059-20 (PMC7773998; doi:10.1128/mBio.03059-20)
Supplement: TABLE S4 [file mBio.03059-20-st004.pdf]

**TABLE S4: Sequenced BSJs from RatPyV2**

| RatPyV2 BSJ Start (nucleotide number) | RatPyV2 BSJ End (nucleotide number) | Strand | BSJ reads | RPM |
|---|---|---|---|---|
| 561 | 3308 | + | 8 | |
| 561 | 3856 | + | 4 | |
| 786 | 1266 | - | 3 | |
| 850 | 4468 | - | 3 | |
| 1653 | 1998 | - | 3 | |
| 4112 | 4468 | - | 11 | 0.001 |

Tissue= RatPyV2 infected parotid gland from an X-SCID Rat, GenBank ID KX574453.1
